# Toxic effect and inability of L-homoserine to be a nitrogen source for growth of *Escherichia coli* resolved by a combination of *in vivo* evolution engineering and omics analyses

**DOI:** 10.3389/fmicb.2022.1051425

**Published:** 2022-12-13

**Authors:** Ceren Alkim, Daniele Farias, Julie Fredonnet, Helene Serrano-Bataille, Pauline Herviou, Marc Picot, Nawel Slama, Sebastien Dejean, Nicolas Morin, Brice Enjalbert, Jean M. François

**Affiliations:** ^1^Toulouse Biotechnology Institute (TBI), Université de Toulouse, CNRS, INRA, INSA, Toulouse, France; ^2^Toulouse White Biotechnology Center (TWB), UMS-INSA-INRA-CNRS, Toulouse, France; ^3^Institut de Mathématique Toulouse, Toulouse, France

**Keywords:** *Escherichia coli*, microbial physiology, L-homoserine, genetic regulation, transcriptomics, evolutionary engineering

## Abstract

L-homoserine is a pivotal intermediate in the carbon and nitrogen metabolism of *E. coli.* However, this non-canonical amino acid cannot be used as a nitrogen source for growth. Furthermore, growth of this bacterium in a synthetic media is potently inhibited by L-homoserine. To understand this dual effect, an adapted laboratory evolution (ALE) was applied, which allowed the isolation of a strain able to grow with L-homoserine as the nitrogen source and was, at the same time, desensitized to growth inhibition by this amino acid. Sequencing of this evolved strain identified only four genomic modifications, including a 49 bp truncation starting from the stop codon of *thrL*. This mutation resulted in a modified *thrL* locus carrying a thrL* allele encoding a polypeptide 9 amino acids longer than the *thrL* encoded leader peptide. Remarkably, the replacement of *thrL* with thrL* in the original strain MG1655 alleviated L-homoserine inhibition to the same extent as strain 4E, but did not allow growth with this amino acid as a nitrogen source. The loss of L-homoserine toxic effect could be explained by the rapid conversion of L-homoserine into threonine *via* the thrL*-dependent transcriptional activation of the threonine operon *thrABC*. On the other hand, the growth of *E. coli* on a mineral medium with L-homoserine required an activation of the threonine degradation pathway II and glycine cleavage system, resulting in the release of ammonium ions that were likely recaptured by NAD(P)-dependent glutamate dehydrogenase. To infer about the direct molecular targets of L-homoserine toxicity, a transcriptomic analysis of wild-type MG1655 in the presence of 10 mM L-homoserine was performed, which notably identified a potent repression of locomotion-motility-chemotaxis process and of branched-chain amino acids synthesis. Since the magnitude of these effects was lower in a *ΔthrL* mutant, concomitant with a twofold lower sensitivity of this mutant to L-homoserine, it could be argued that growth inhibition by L-homoserine is due to the repression of these biological processes. In addition, L-homoserine induced a strong upregulation of genes in the sulfate reductive assimilation pathway, including those encoding its transport. How this non-canonical amino acid triggers these transcriptomic changes is discussed.

## Introduction

The metabolic pathway leading to L-homoserine is very well established genetically and biochemically in model and industrially relevant microorganisms such as *Escherichia coli* (*E. coli*), *Corynebacterium glutamicum* (*C. glutamicum)* and the yeast *Saccharomyces cerevisiae* (*S. cerevisiae*). This non-essential chiral amino acid is a branch point metabolite for the synthesis of essential amino acids threonine and methionine. It is formed from aspartate through a three-reaction step catalysed by aspartate kinase (AK), aspartate semi-aldehyde dehydrogenase (ASD) and L-homoserine dehydrogenase (HDH) (Cohen, 1983). A peculiarity of *E. coli* is that the first reaction is catalysed by three isoenzymes termed AKI, II and III. In addition, two of these aspartate kinases, namely AKI and AKIII encoded by *thrA* and *metL* are bifunctional enzymes as they also carry the HDH activity (Cohen, 1985). The connection with the carbon central pathway takes place at the level of the TCA intermediate oxaloacetate (OAA), which is converted into aspartate by the aspartate–glutamate transaminase encoded by *aspC*. The metabolic engineering of *E. coli* for the production of L-homoserine has been recently studied by different research groups, leading to titer of 35 to 110 g/l and yield on glucose ranging from 0.35 to 0.64 g/g in aerobic batch fermentation conditions (Li et al., 2017; Liu et al., 2020; Mu et al., 2021; Vo and Park, 2022). Intriguingly, these works did not report any toxic effect of L-homoserine on growth, perhaps because the engineered strains were optimised for efficiently export this non-canonical amino acid. Recent works also shed light on engineering of non-natural pathways in which L-homoserine is a precursor in the synthesis of 1,3-propanediol (PDO)(Chen et al., 2015; Zhong et al., 2019), 2,4 dihydroxybutyric acid (DHB) (Walther et al., 2018), as well as a key intermediate in two cyclic synthetic pathways devoted to C1 assimilation, which are CO_2_ (Bouzon et al., 2017) and methanol (He et al., 2020).

Despite its central position in the carbon-nitrogen metabolic network, it is very surprising that L-homoserine cannot be used as a nitrogen source of *E. coli*, although this non-canonical amino acid can be transported by the branched-chain amino acid LIV-1 and LS systems (Templeton and Savageau, 1974) or by the threonine importer encoded by *tdcC* (Sumantran et al., 1990). In addition, millimolar concentration of L-homoserine causes growth inhibition and two distinct mechanisms of action have been considered. On the one hand, L-homoserine could alter the leucine-tRNA synthetase-dependent fidelity of protein synthesis by competing with leucine for tRNA aminoacylation (Karkhanis et al., 2007). On the other hand, it was shown that the activity of NADP^+^-glutamate dehydrogenase, which catalyses the main ammonium assimilation reaction in *E. coli* was 50% inhibited by 10 mM L-homoserine (Kotre et al., 1973). However, these two proposed mechanisms are not really consistent with the finding that the growth of *E. coli* was impaired by less than 10 mM L-homoserine [(Li et al., 2016), our unpublished data]. Besides, L-homoserine toxicity was also reported in *Mycobacterium tuberculosis* (Obarr and Everett, 1971), yeasts (Kingsbury and McCusker, 2010) and mammalian cells (Rees et al., 1994). In *M. tuberculosis*, it has been proposed that L-homoserine toxicity is due to 2-amino-n butyric acid, which is formed by the transfer of the NH_2_ group of L-homoserine to 2-ketobutyrate, the latter coming from threonine degradation by threonine deaminase. However, the molecular targets of 2-amino-n butyric acid have not been identified (Obarr and Everett, 1971). In the yeasts *Saccharomyces cerevisiae* and *Candida albicans,* L-homoserine toxicity was found in strains that are defective in enzymes converting L-homoserine into threonine (deletion of *THR1* or *THR4* encoding L-homoserine kinase or threonine synthase) (Kingsbury and McCusker, 2010) or in engineered yeast cells for high production of threonine (Farfan and Calderon, 2000). It was shown that this toxicity can be alleviated by activation of the proteasome or the ubiquitin pathway and antagonized by threonine (Kingsbury and McCusker, 2010), leading to the hypothesis that L-homoserine acts as a threonine analogue that inhibits a particular threonine-sensitive protein required for growth. Overall, these data indicate that L-homoserine toxicity appears to be ubiquitous but its mechanism of action seems to differ between these microbial systems.

In this work, we used an *in vivo* evolutionary engineering strategy to isolate an evolved strain capable of growing on a mineral glucose medium with L-homoserine as the sole nitrogen source and applied omics technologies (transcriptomics and metabolomics) to reveal the mechanism by which L-homoserine is toxic for growth of *E. coli*.

## Materials and methods

### Strains and growth media and culture conditions

Strains of *E. coli* used in this study are listed in Supplementary Table S1. Unless otherwise stated, *E. coli* strain MG1655 has been used throughout this study as the recipient strain for genetic construction and will be referred to as *E. coli* wild type (WT). LB medium (0.5% yeast extract, 1% tryptone, 0.5% NaCl) was used for strain engineering and recombinant plasmids cloning. When required, antibiotic was added to the medium at the following concentration: kanamycin, 50 μg/ml; streptomycin, 100 μg/ml; chloramphenicol, 25 μg /mL; ampicillin, 100 μg/ml. Growth experiments was carried out in M9 mineral medium buffered at pH 7.0 with MOPS 100 mM as described in Trichez et al., (2018) containing unless otherwise stated glucose (2% w/v) at 37°C on a rotary shaker (Infors Multitron) set at 200 rpm and monitored spectrophotometrically at OD 600 nm (OD_600_).

For L-homoserine growth assay and toxicity test, various *E. coli* strains were first cultured overnight at 37°C in 2 ml M9 medium in 15-mL falcon tube from a single colony from a culture on LB solid medium. Pre-cultures were harvested, washed twice with M9 medium, then 200 μl of M9 culture medium containing L-homoserine at indicated concentration in the corresponding figures were inoculated with bacteria cells at OD_600_ 0.15 in triplicate in 96-well microplates, which were incubated at 30°C in a Biotek, using Synergy HTX Multi-mode reader, allowing continuous monitoring of growth at OD_600_, or with microplate fixed in Infors shaker at 37°C.

### Adaptation of *Escherichia coli* to growth on L-homoserine by *in vivo* evolutionary engineering

The GM3 automated technology for evolving microbial populations in continuous culture developed by Marlière & Mutzel Mutzel and Marliere, (2000) and commercialized by Altar^1^ has been employed to adapt *E. coli* MG1655 to high concentration of L-homoserine according to the following experimental design. At first, the cell population of wild type MG1655 was cultivated in a M9 medium containing 0.2% (w/v) glucose and 10 mM L-aspartate (permissive medium) in a turbidostat mode until growth rate was stabilized. Then, the culture was subjected to a medium-swap process, which corresponded to pulse addition of a restrictive medium (M9 containing 0.2% glucose and 10 mM L-homoserine), at a constant growth rate of 0.2 h^−1^. After adaptation into the restrictive medium, another run in turbidostat mode was applied to significantly improve the specific growth rate of the cell population. Genetic stability of 10 independent clones randomly selected from adapted cellular population was carried out by 5 successive passages into LB medium before retesting L-homoserine sensitivity of these independent clones in M9 medium.

### DNA manipulation

Deletion of *thrL* in MG1655 was performed using the phage transduction method adapted from Miller (1992). The phage lysate was prepared from the *ΔthrL* strain of Keio collection (Baba et al., 2006). Positive clones were selected on kanamycin-containing LB agar plates and verified by PCR analysis. The Kan^R^ cassette was removed from the genome by expressing FLP recombinase from the pCP20 plasmid and excision of the cassette was verified by PCR using locus specific primers (see Supplementary Table S2). The replacement of wild type *thrL* by mutated thrL* in MG1655 strain was carried out using CRISPR/Cas9 method according to Li et al., (2015). To construct gRNA plasmid, a set of primers was used to PCR amplify the pGRB backbone (see Supplementary Table S3). The 20 bp spacer sequence specific for gene target was design with Atum tools.^2^ An overlap PCR allowed self-ligating PCR products to obtain gRNA plasmid. Donor dsDNA purchased from Genewiz, corresponded to 300 bp arms at the 5′ and 3 end of thrL* gene. PCR amplification of donor dsDNA and purification on agarose gel was realized before used for integration. Integration was done in MG1655 which was performed by electroporation as described in Wang et al., (2009), with 400 ng donor dsDNA (or 1.0 μM ssDNA) and 100 ng gRNA plasmid. Bio-Rad MicroPulser was used for electroporation (0.2 cm cuvette, 2.50 kV). Upon electroporation, cells were immediately mixed with 0.5 ml LB and recovered for 3 h prior to plating. For plasmid curing, colonies were inoculated in LB containing 0.5 mM IPTG and kanamycin at 50 μg/ml and cultivated for 6 to 8 h or overnight at 30°C. After 2–3 passages in LB medium supplemented with IPTG and kanamycin, colonies no longer resistant to spectinomycine were selected. These colonies were cultivated in LB medium at 42°C to eliminate Cas9 plasmid. Then, cultures after plasmid curing were streaked and colonies were tested for kanamycin sensitivity.

### Cloning, expression, and production of *thrL* and thrL* encoding polypeptides

The synthetic wild type *thrL* was purchased from Eurofins (France) carrying the *Bam*HI and *Hind*III sequences at its 5′ and 3′ ends for cloning into pET-28a(+) digested with *Bam*HI and *Hind*III using the NEBuilder HiFi DNA assembly kit (NEB BioLabs). The same operation was performed for thrL* allele except that the sequence of this mutant gene was obtained by amplification from the chromosomal DNA of the L-homoserine-adapted strain 4E with a high-fidelity polymerase (NEB BioLabs) using the forward and reverse primers listed in Supplementary Table S2. The constructed plasmids were sequenced before transformation of *E. coli* strain DH5α cells (NEB Biolabs). One colony from each of the transformant was inoculated in 5 ml LB medium containing 50 μg/ml kanamycine (LB + KAN) and grown overnight at 37°C. This pre-culture was then used to inoculate 200 ml LB + KAN in 1-L baffled flasks at an initial OD_600_ of 0.05. The flasks were incubated at 37°C with 200 rpm and IPTG was added at a final concentration of 0.5 mM when the culture reached OD_600_ of 0.6. They were cultivated for an additional 3 h at 37°C and then harvested by centrifugation at 4°C for 15 min at 4000 *g*. Cell pellets were stored at −20°C for further protein purification. His SpinTrap TALON column (GE Healthcare) was used for purification of his-tagged ThrL and ThrL* proteins. Proteins were separated by Sodium Dodecyl Sulfate-Polyacrylamide gel electrophoresis (SDS-PAGE) in Tris-Tricine 16.5% gels (Bio-Rad) by using 10 times diluted Tris/Tricine/SDS running buffer (Bio-Rad) for 40 min at 160 V. The proteins in the gel were transferred onto 0.2 μm nitrocellulose membrane by using Trans-Blot Turbo transfer system (Bio-Rad). The membrane was incubated with anti-polyhistidine-peroxidase antibody (Merck) diluted 10,000 times that can react with His-tagged fusion proteins. Clarity Western ECL Substrate solution (Bio-Rad) was used for immunological detection.

### Whole genome sequence analysis

The sequencing of *E. coli* clones was carried out by the GeT-Biopuces platform^3^ using the Ion Torrent/ThermoFisher Technology. The quality of extracted DNA was evaluated by gel electrophoresis (1% agarose) and the nucleic acid concentrations were quantified by Qubit™ (Thermo scientific). The libraries were performed from 100 ng of DNA and amplified using NEBNext® Fast DNA Fragmentation & Library Prep and Ion Xpress™ Barcode Adapters according to the manufacturer’s recommendations (New England Biolabs & ThermoFisher). The obtained libraries were quantified with the Qubit™ (dsDNA HS Assay Kit) and the Bioanalyzer 2,100 (Agilent-DNA1000 kit). Using the Ion 520 & 530 OT2 kit (Thermofisher), each library were pooled together and sequenced (210-270pb oriented single end) on an Ion 520 chip on the Ion Torrent S5 using according to manufacturer’s instructions. All the raw reads were processed with Torrent_Suite (v 5.0.4): they were aligned with tmap (v 5.0.13) against the reference sequence of *E. coli* (Escherichia_coli_str_k_12_substr_mg1655.ASM584v2), then the variants were search with TorrentVariantCaller (tvc v 5.0–13, with parameter of germline_low_stringency_pgm_520_530, TS version: 5.0).

### DNA microarrays experimental procedure and data treatment

An overnight pre-culture of *E. coli* strains in M9 medium was inoculated in 0.3 l shake flasks containing 50 ml of the same medium (initial OD_600_ of ∼0.2) in the absence or presence of 10 mM L-homoserine. Cultures were carried at 37°C at 200 rpm until OD_600_ reached ~1.0 to 1.5. At this time, 5 times one milliliter of culture was pipetted in microcentrifuge tubes and centrifuged at 10,000 *g* for 2 min. The cell pellets were quickly washed once with 1 ml cold water, resuspended and quickly centrifuged at as above. After draining remaining water on a towel, the tubes were thrown in liquid nitrogen and stored at −80°C until use. This procedure was repeated three time with three biological independent cultures and for each strain. RNA were extracted frozen cell pellets using the QIAGEN RNeasy Mini Kit RNA, quantified by NanoDrop (Thermo) and its quality control was validated on Bioanalyzer (Agilent Technologies). Only RNA samples with a RIN (RNA Integrity Number) higher than or equal to 9.00 were chosen for microarray analysis. The RNA were converted to cDNA using the Low Input Quick Amp labeling kit (Agilent) with dCTP(CY3) labelling. The labeled cDNA was hybridized on *E. coli* Gene Expression Microarrays (8 × 15 K, Agilent) following the Agilent One-Color Microarray-Based Gene Expression Analysis Protocol. The slides were scanned on a Tecan scanner MS200 and analyzed by Feature Extraction V.11.5.1.1. Signals of each probe set were filtered according to the coefficient of variations with a cut-off value of 50%. Significant changed expression was acquired by moderated *t*-test with a *value of p* of <0.05 and a fold-change cut-off value of 2.0. Benjamini-Hochberg correction was performed (Hochberg and Benjamini, 1990). Further details on transcriptomic analyses is reported in an accompanying paper to be submitted to Data in Brief.

### Data treatment and statistical analysis

The package mixOmics^4^ was used for statistical treatment of the transcriptomic data sets. The tool has its own tutorial and can be easily used, for a nearly inexperienced R user, through the web site at http://mixomics.org/. Specific functions for genes ontology, pathways tools, etc. were searched using tools available at the ECOCYC website^5^ (Keseler et al., 2017). Clustered heat maps of transcriptomic analysis were performed using the Interactive Clustered map builder freely available at https://build.ngchm.net/NGCHM-web-builder/ (Ryan et al., 2019).

## Results

### The *Escherichia coli* strains cannot utilize L-homoserine as a nitrogen source and moreover this non canonical amino acid is toxic for growth

**Figure 1:**
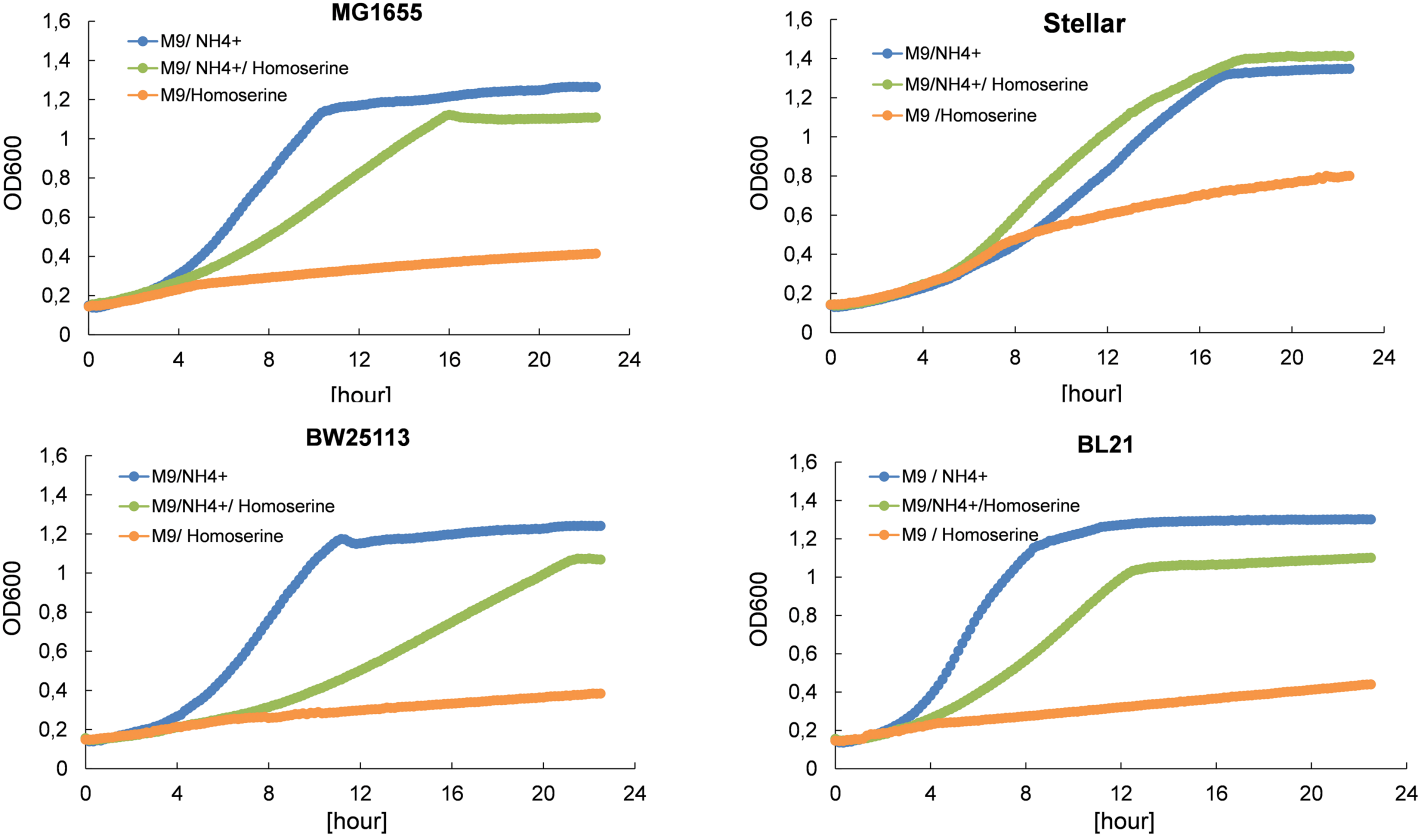
L-homoserine is not a nitrogen source for growth of *E. coli* and causes growth inhibition when added to a mineral medium containing another source of nitrogen (ammonium ions). The growth was carried out in M9 medium buffered at pH 7.0 with 100 mM MOPS containing 0.4% (w/v) glucose at 30°C in a Biotek microplate reader. L-homoserine was added at 15 mM. Growth curves for each strain and each condition are the mean of three technical replicates.

Although L-homoserine has a pivotal position in the carbon-nitrogen metabolic network, it cannot be used as a nitrogen source for growth of *E. coli* as shown in Figure 1. Moreover, the growth of various *E. coli* strains on a mineral M9 medium with ammonium sulfate as nitrogen source was strongly inhibited by millimolar concentration of L-homoserine, although this inhibitory effect was quantitatively different between strains (see Supplementary Figure S1). Table 1 reports the concentration of L-homoserine that resulted in 50% reduction of the growth rate for different *E. coli* strains. The *E. coli* strain JS200 exhibited the highest sensitivity with a IC_50_ in the range of 3 mM, whereas strain BW25113 used as the recipient to generate the Keio collection of single gene mutants (Baba et al., 2006) had similar sensitivity as MG1655 to L-homoserine. In contrast, *E. coli* strains BL21 commonly used for protein production (Phue et al., 2008) were less sensitive, showing a 50% growth rate reduction at a concentration above 10 mM of L-homoserine.

**Table 1:** Toxicity of L-homoserine on different *E. coli* strains as determined by the concentration at which this compound reduced by 50% the maximal growth rate (IC_50_).

***E. coli* strain**	**IC**_**50**_ * (mM)
MG1655	3.3 ± 1.8
BW25113	3.6 ± 1.9
Stellar	8.5 ± 2.9
NICO21	6.0 ± 1.9
BL21	13 ± 2.9
JS200	2.6 ± 0.6
NEBα	>15
BW25113 *ΔlivJ*	4.6 ± 1.9
BW25113 *ΔtdcC*	3.9 ± 1.2
MG1655 Δ*thrl*	5.8 ± 1.7

Growth was carried out in M9 medium buffered at pH 7.0 with 100 mM and containing 0.4% glucose and IC_5O_ corresponded to the value at which growth rate (μ) was half of the μ_max_ in the absence of L-homoserine. Data are the mean ± SD of three independent biological cultures.

Since L-homoserine can be transported in *E. coli* through either the branched-chain amino acid LIV-I and LS systems (Templeton and Savageau, 1974) encoded by *livJHMGF* and *livKJMGF* (Adams et al., 1990; Koyanagi et al., 2004) or by the threonine importer encoded by *tdcC* (Sumantran et al., 1990; Liu et al., 2020), we examined whether ablation of these transporter systems alleviated this inhibition. Quite surprisingly, the sensitivity of strain BW25113 to L-homoserine was little affected by the deletion of *tdcC*, *livJ* or *livK* (Table 1 and in Supplementary Figure S1). Moreover, threonine or isoleucine (10 mM), which are transported by the same transporters, could not antagonize the effect of L-homoserine (data not shown). Taken together, these data indicate that either the inhibitory effect of L-homoserine occurs at the cell wall and does not require its uptake or that the uptake is carried out by nonspecific transporters, which could be possible since the affinity of the reported transporters for L-homoserine is in the range of 5 to 10 μM (Templeton and Savageau, 1974), while a 1000-fold higher concentration of L-homoserine was used in this study.

### Desensitization of *Escherichia coli* to L-homoserine by *in vivo* evolutionary engineering

Adapted laboratory evolution (ALE) is a powerful strategy widely used to evolve microbial cells with a desired characteristic, such as the resistance to a toxic molecule or to overcome a metabolic bottleneck that penalizes growth (Mavrommati et al., 2021). We therefore used the automated GM3 device patented by Mutzel and Marliere (2000; see Footnote 1) in order to adapt *E. coli* to grow on a mineral glucose medium with L-homoserine as the sole nitrogen source. The ALE was initiated by adapting strain MG1655 in a permissive medium (M9 medium with 0.2% glucose and 10 mM aspartate) following a turbidostat mode for 16 days until the growth rate was stabilized at around 0.65 h^−1^ (see Supplementary Figure S3A). Then, the cell population was subjected to a medium-swap mode that consisted to cultivation in a permissive medium (M9 /0.2% glucose with L-aspartate 10 mM) with pulse addition of a restrictive medium (M9 / 0.2% glucose with 10 mM L-homoserine) at increasing percentage of the culture volume while keeping constant the growth rate at 0.2 h^−1^. Adaptation of the cell population to the restrictive medium was achieved when the pulse addition reached 100%, which required approximately 16 days (see Supplementary Figure S3B). This was followed by a turbidostat culture mode for 16 days to further improve the growth rate on this medium. Overall, this automatized ALE took around 48 days (Supplementary Figure S3) to yield a cells population adapted to grow at about 0.42 h^−1^ in a M9 glucose medium containing 10 mM L-homoserine. Samples of this final population were streaked on LB-plates and 10 individual colonies were picked randomly and cultivated 5 times successively in LB medium before retesting their growth in a M9 medium containing 10 mM L-homoserine. We found that 90% of the isolated colonies grew very well on this medium while the unevolved strain did not grow, indicating that the phenotypic trait was genetically stable. One of them, which we referred to strain 4E was retained for further genomic, transcriptomic and metabolomic characterization.

### Identification of genomic modifications in L-homoserine adapted *Escherichia coli* clones

At the three stages along the *in vivo* evolutionary engineering experiment, clones were isolated and subjected to whole genome sequencing to identify the genetic modifications that have occurred during the ALE experiment. As reported in Table 2, only 4 genomic modifications were identified in the final evolved strain 4E, with two of them, namely *rpoB* and *alaC* genes, already present in the aspartate-adapted (MG-Asp) clone. For *alaC* which encodes one of the three L-alanine - α-ketoglutarate transaminase (Kim et al., 2010), the single G to C change at position +234 bp from the start codon leads to the replacement of arginine by cysteine at position 78 (R78C). Although this amino acid residue does not take part in the catalytic active site (Pena-Soler et al., 2014), we investigated the kinetic properties of this AlaC^R78G^ variant and found that it displayed a 2-fold increase in V^max^ and a threefold reduction of K_M_ for glutamate, leading to a 5 fold increase in the catalytic efficiency (k_cat_/K_M_) on this substrate (Supplementary Table S4). This higher activity on glutamate may contribute to increased growth rate on L-aspartate because it could enhance the recycling of α-ketoglutarate which is needed for assimilation of L-aspartate into TCA cycle. The other mutation was in the *rpoB* gene that encodes the β subunit of RNA polymerase. The mutation H526Y is located in a region where elongation activity can be positively affected (Landick et al., 1990), providing a fitness gain (Rodriguez-Verdugo et al., 2016) to the cell.

**Table 2:** Genomic modifications of *E. coli* clones adapted first on aspartate in a turbidostat mode (MG_Asp), then to L-homoserine by applying a medium swap (MG_Hms) and finally to L-homoserine in a turbidostat mode (HMSRC4E).

**Gene**	**Function**	***E. coli* clones**	**SNP/Del**	**Relative position**	**Mutation type**	**AA change**
**alaC**	alanine-2-oxoglutarate aminotransferase	MGAsp; MGHms, HMSRC4E	G- > C	24,98,063	CGG- > GGG	R78G
**rpoB**	beta-subunit of RNA polymerase	MGAsp; MGHms; HMSRC4E	C- > T	4,182,820,	CAC - > TAC	H526Y
**thrL**	a 21 amino acid long peptide (peptide leader) controlling *thrABC* operon	MGHms;HMSRC4E	Del	253 to 294	mutation at the stop codon	Potential addition of 9 aa
**purL**	Formylglycinamide ribonucleotide amidotransferase	HMSRC4E	G- > C	26,94,932	CAG- > AAC	Q211H

*Verification of sequence at the thrL locus. SNP, Single Nucleotide Polymorphism; Del, deletion; AA change, amino acid change.

The two remaining genomic modifications, namely on *purL* and *thrL* genes were specifically identified in L-homoserine adapted strain 4E (Table 2). The *purL* gene encodes the phosphoribosylformylglycinamide synthetase that catalyses the fourth step in *de novo* purine biosynthesis. It converts 5′-phosphoribosyl-*N*-formylglycineamide (FGAR) to 5-phosphoribosyl-*N*-formylglycineamidine (FGAM) in the presence of glutamine and ATP. The single G to T transition in *purL* led to the replacement of a glutamine by histidine at position 211 (Q211H) in PurL. This mutation is located in the linker domain of the PurL protein, which is also in the vicinity of the ATP binding site located in the FGAR domain (Anand et al., 2004). Assuming that this mutation causes the loss of PurL activity, a phenotype associated to this loss is a lack of growth on a glycerol synthetic medium (Joyce et al., 2006), which we did not observe with the strain 4E (data not shown). Thus, the occurrence of this single mutation in *purL* in L-homoserine-adapted strain 4E remained unexplained.

**Figure 2:**
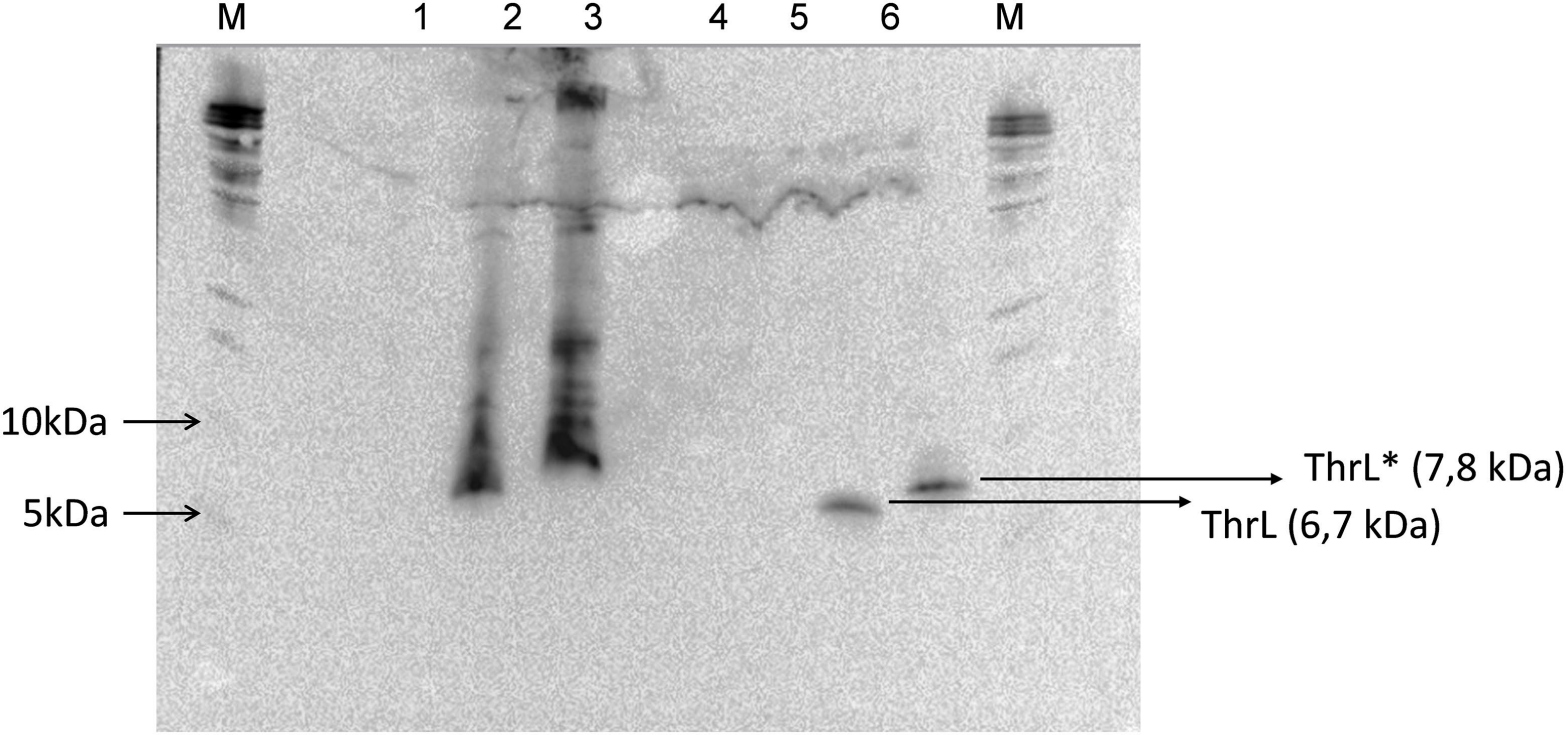
Western blot analysis of His-tagged ThrL and ThrL* protein with anti-His antibodies. Lanes 1, 2 and 3 corresponds to cell lysate of *E. coli* transformed with pET28(+), pET28(+) carrying *thrL* and pET28(+) carrying thrL*. Lanes 4, 5 and 6 are same samples after purification on an affinity Co^2+^ column. M means Marker.

At variance to single mutations described so far, the genomic modification at *thrL* locus corresponded to a 49-bp truncation that starts at the stop codon of the *thrL* gene, which codes for the leader peptide that controls the expression of the *thrABC* operon by an attenuation mechanism (Gardner and Smith, 1975), and encompassed 28 bp of the transcription terminator site (*thr* attenuator, also known as Rho-independent terminator) (Supplementary Figure S4). Interestingly, this genomic modification could have changed the reading frame of *thrL,* resulting in a size extension of the encoded polypeptide from 21 to 30 amino acid residues. To verify this assumption, the wild type *thrL* and the mutated allele, named thrL*, were cloned in pET28^+^ under the Tac promoter, transformed in *E. coli* BL21 and expressed *in vivo* after addition of IPTG. Lysates from these cells were run on a SDS PAGE and the gel was blotted on a nitrocellulose membrane to carry out Western blot analysis with the anti-His antibodies that should react with the His-tag of the produced ThrL and Thrl* protein. Results of this experiment shown in Figure 2 supported our assumption, since a band at the expected size of 6.7 kDa for the normal ThrL (*ie* 21 aa +6 histidine +27 aa for the spacer in the pET28+ vector) and at 7.8 kDa for the ThrL* variant (30 aa +6 histidine +27 aa of the spacer) that specifically cross-reacted with the anti-His antibodies were visible in both the cell lysates and after purification on Co^2+^ affinity column.

### Replacement of *thrL* gene by thrL* allele alleviated L-homoserine toxicity but does not allow growth with this amino acid as a nitrogen source

**Figure 3:**
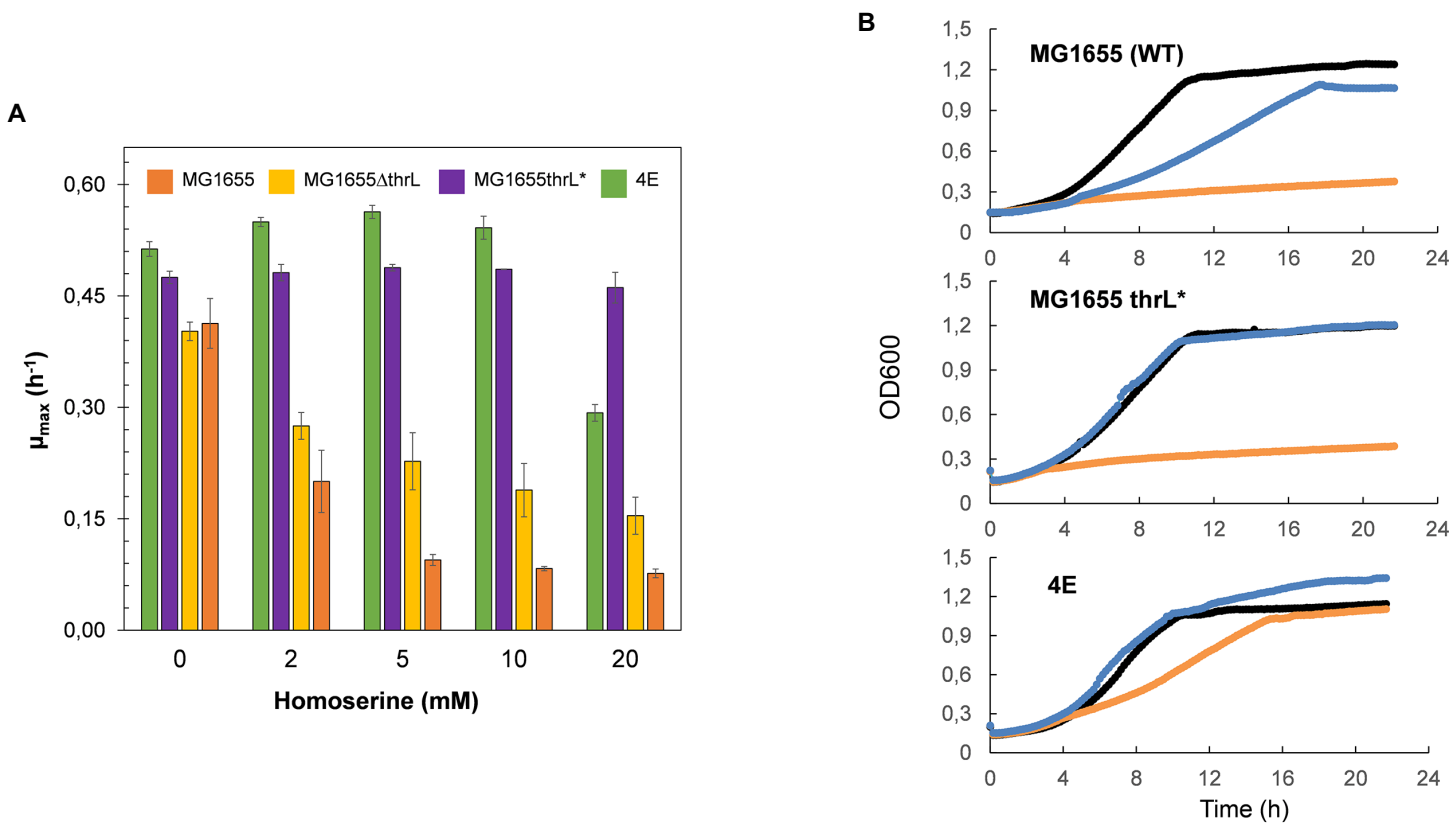
Replacement of *thrL* by thrL* in wild type MG1655 alleviated L-homoserine toxicity but does not allow growth with this amino acid as sole nitrogen source. In **(A)** effect of L-homoserine on maximal growth rate (μ) of MG1655, MG1655 *ΔthrL* MG1655 thrL* and 4E strain. In **(B)** growth of MG1655, MG1655 *ΔthrL*), MG1655 thrL* and 4E on M9 with NH4^+^ (black line), with NH4 and 10 mM homoserine (blue line) or with 15 homoserine (orange line). Data are the mean of three technical replicates.

Banking that the dramatic genomic modification at the *thrL* locus could be implicated in the effects of L-homoserine on *E. coli* growth, this locus spanning +160 to +320 bp on the MG1655 genome was replace by thrL* locus using the CRISPR-Cas9 method (Bassalo et al., 2016). As can be seen in Figure 3A, this replacement alleviated L-homoserine toxicity to a level even better than that of the evolved strain 4E. This release of toxicity was specific to this allele since the loss of *thrL* function in MG1655 only resulted in a twofold reduction of L-homoserine toxicity as compared to the wild type strain. In contrast, a MG1655 carrying thrL* allele was unable to growth on a M9 medium with L-homoserine as sole nitrogen source (Figure 3B). Altogether, these results indicate that the inability of *E. coli* to use L-homoserine as nitrogen source is not due to the toxicity effect of this compound, and therefore, these two features of L-homoserine likely implicate different mechanistic pathways.

### Exometabolome analysis provided clues as to how *Escherichia coli* overcomes L-homoserine toxicity

**Figure 4:**
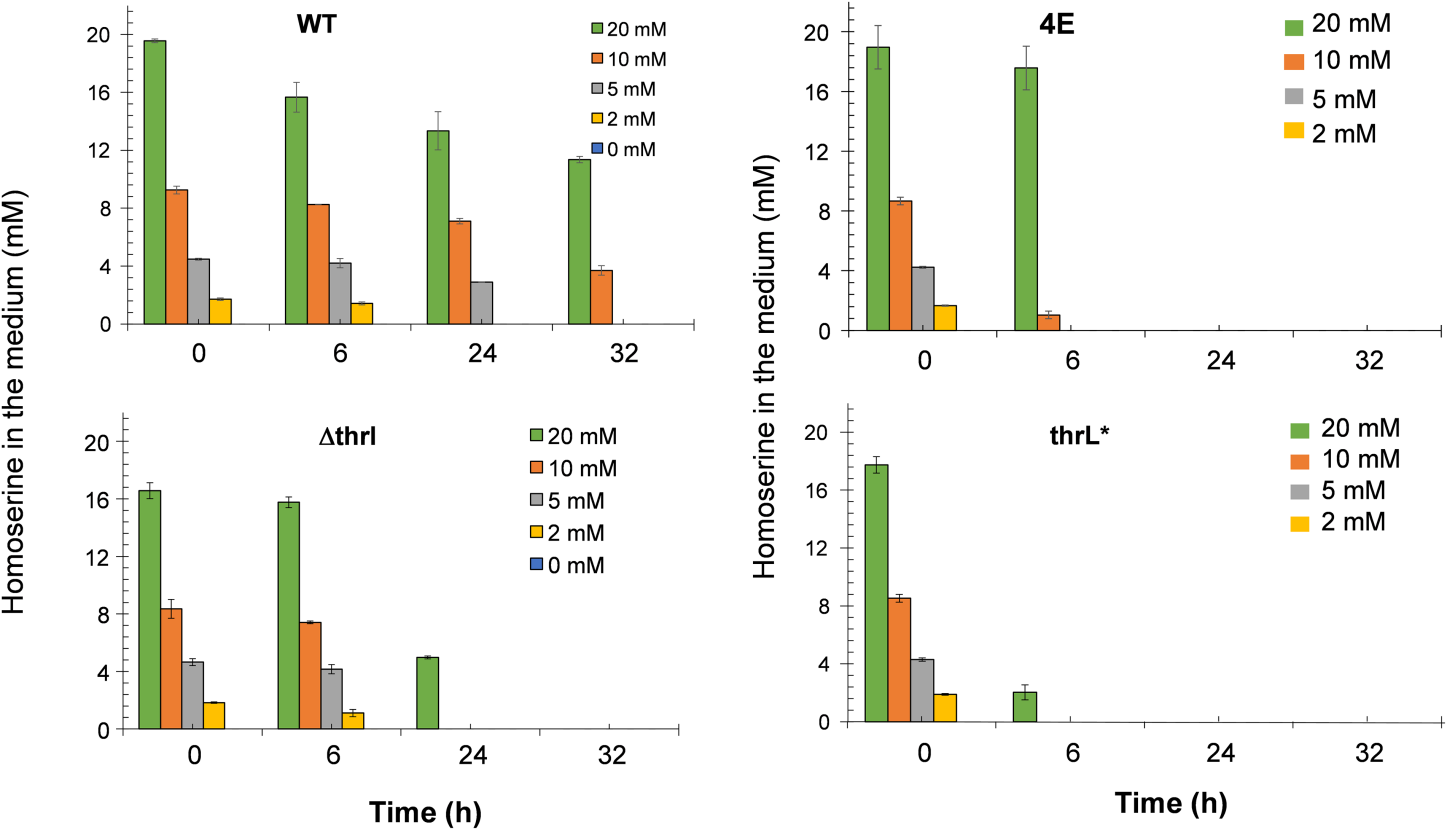
Fate of exogenous L-homoserine in MG1655 (WT), MG1655 deleted for *thrL* (ΔthrL), MG1655 in which *thrL* was replaced by thrL* (thrL*) and homoserine-adapted strains (4E) during batch growth in M9 mineral medium with 2% (w/v) glucose at 37°C. Data are the mean of three biological replicates and the SD is represented by bars on the histograms.

**Figure 5:**
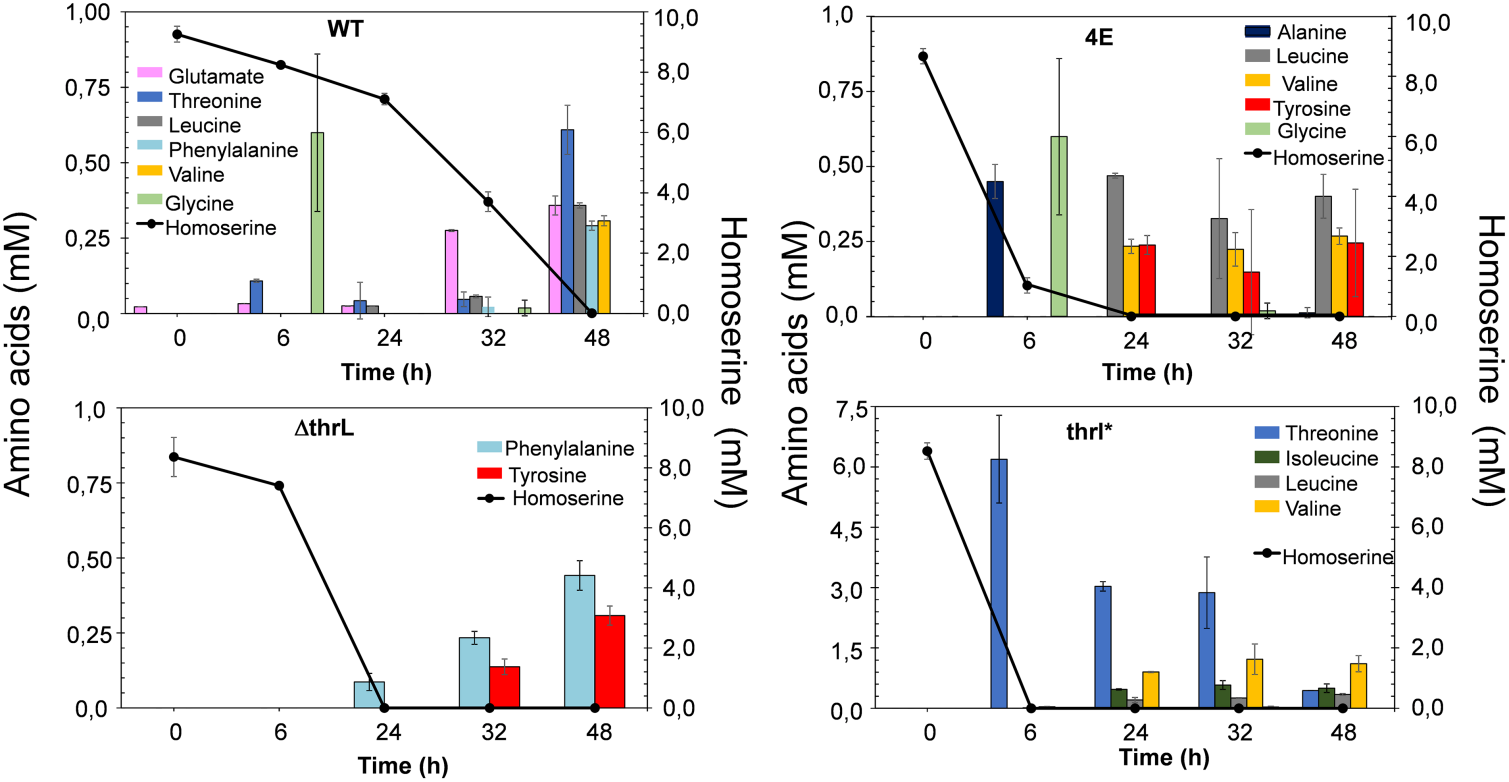
Exometabolome in MG1655 (WT), MG1655 deleted for *thrL* (ΔthrL), MG1655 in which *thrL* was replaced by thrL* (thrL*) and homoserine-adapted strains (4E) during growth in M9 medium buffered at pH 7.0 by MOPS 100 mM and containing 2% (w/v) glucose and in the presence of 10 mM L-homoserine. Data are the mean of three biological replicates, with SD represented by a bar on the histograms. Notice the difference in the scale for amino acids level in the exometabolome pattern of strain thrL*.

The alleviation of L-homoserine toxicity upon replacement of *thrL* by thrL* suggested the activation of a detoxification process that could be dependent on this mutant gene. A direct indication of this process can be obtained by following the fate of L- homoserine during growth. As can be seen in Figure 4, L-homoserine depletion was extremely slow in wild type MG1655 and was approximately 2 to 3 fold faster in the *Δthrl* strain. These data show that L-homoserine, while toxic to growth, is slowly metabolized and that its faster metabolism in ΔthrL strain is consistent with a twofold lower sensitivity of this strain to L-homoserine compared to the wild type (see Figure 1). As expected, L-homoserine depletion was extremely rapid in the L-homoserine-evolved strain 4E and even more rapid in MG1655 carrying the thrl* mutant allele, with complete depletion approximately 6 to 8 h after the start of growth. These data clearly indicated that the loss of L-homoserine toxicity in these two strains can be explained by the rapid depletion or degradation of this compound. Therefore, the next question to be solved was to identify the compounds resulting from the metabolism of L-homoserine. To this end, we analyzed the culture medium for the presence of potential compounds such as threonine, methionine, 2-oxobutanoate or glycine, as they may originate from degradation pathways of L-homoserine (FC et al., 1996). Figure 5 shows the results from cultures in the presence of 10 mM L-homoserine (data of cultures made without and with 2, 5 and 20 mM L-homoserine can be found in Supplementary Figure S5). It can be seen that the exometabolites profiles were very different between the four strains. The exometabolome of strain MG1655 (WT) showed a transient accumulation of glycine early after the start of the growth that corresponded to max. 30% of L-homoserine consumed and an accumulation of threonine at the end of the growth. However, no products accumulated during the disappearance of L-homoserine in the *ΔthrL* mutant, although the rate of disappearance of this amino acid was two times faster. The rapid depletion of L-homoserine in strain 4E was accompanied by a transient accumulation of L-alanine and glycine that overall corresponded to less than 10% of the L- homoserine consumed. The formation of L-alanine could originate from the reaction catalyzed by the alanine-pyruvate transaminase encoded by *alaC*, as this enzyme was shown to be able to transfer the NH_2_ group of L-homoserine to pyruvate to yield alanine and 2-keto-4-hydroxybutyrate (Bouzon et al., 2017). Interestingly, we reported above that *alaC* in strain 4E harbors a C to G mutation that results in the AlaC^R78G^ variant, but this mutation did not improve the activity on L-homoserine (data not shown). On the other hand, the weak or even absence of glycine suggested that the glycine cleavage system (GCS) encoded by the *gcvTHP* operon (Stauffer et al., 1994) may be activated in this strain.

In contrast to the exometabolome of strain 4E, the disappearance of L-homoserine in MG1655thrL* was accompanied by an accumulation of threonine equivalent to the amount of L-homoserine consumed, and this correlation was found regardless of the concentration of L-homoserine present in the medium (see Supplementary Figure S5). This result suggested an activation of the threonine synthesis pathway dependent on *the thrABC* operon. To validate this suggestion, *thrB* encoding the homoserine kinase was deleted leading to a growth inhibition by L-homoserine of the ΔthrB thrL* mutant almost comparable to strain MG1655 (see Supplementary Figure S6). In summary, analysis of the exometabolite profiles of strains 4E and MG1655thrL* strongly supports the idea that the loss of L-homoserine toxicity is due to its rapid degradation.

### Detoxification to L-homoserine is due to its rapid conversion into threonine

**Figure 6:**
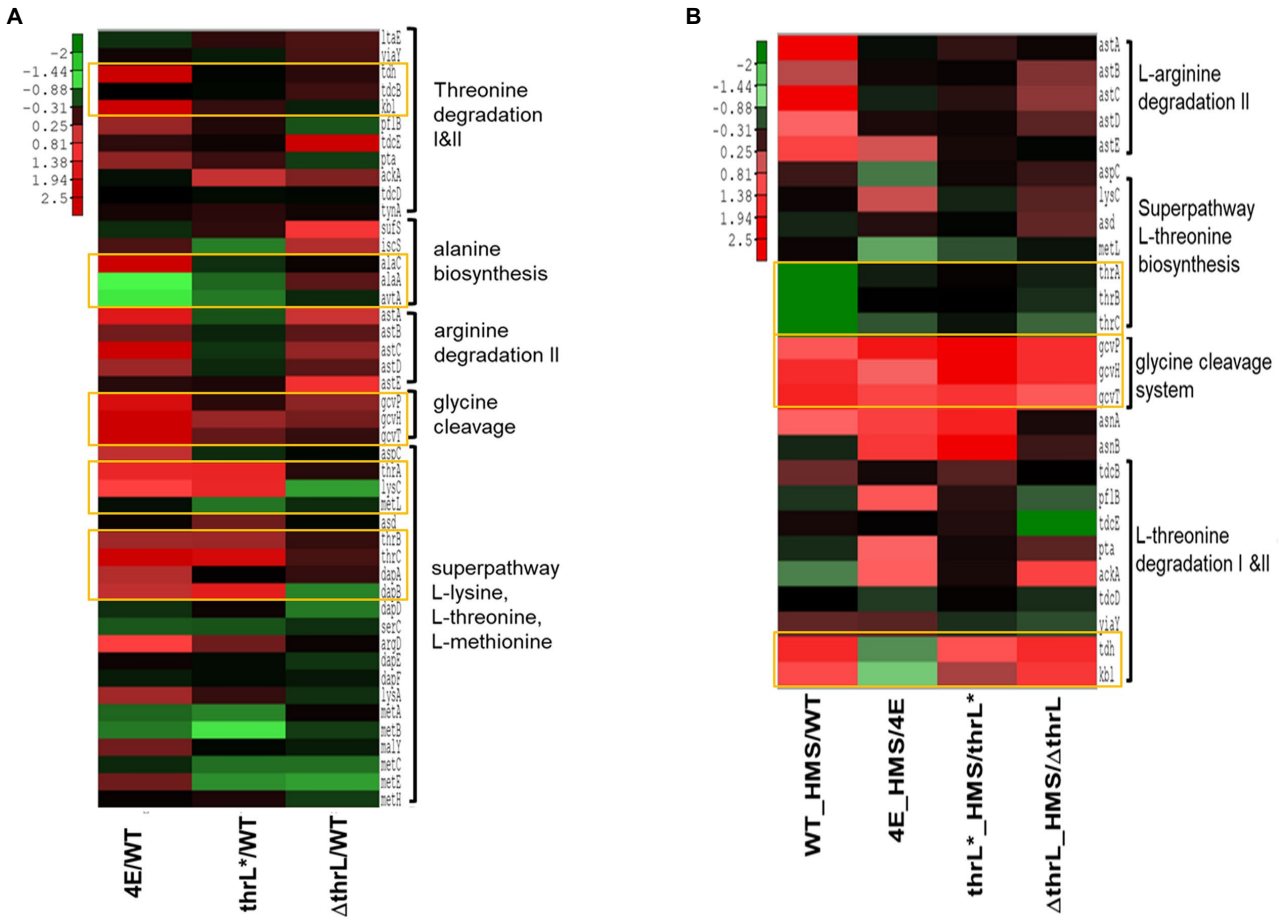
Heat map representation of the expression of genes implicated in threonine, glycine, L-alanine and L-arginine superpathway. In **(A)** is shown expression of these genes in homoserine-adapted (4E), MG1655thrL* (thrL*) and MG1655Δthrl (ΔthrL) strains versus that in WT when cultivated in M9 medium. In **(B)** is shown the expression of these genes in strains 4E, thrL*, *ΔthrL*, and WT in the presence versus the absence of 10 mM L-homoserine. Up- and down-regulated genes (at least 2-fold variation and a *p* value <0.05) were analysed by a clustering method described in Material and Methods. The expression fold of repression (green) or activation (red) of genes is shown by color-coded bar.

To evaluate our assertion that alleviation of L-homoserine toxicity is due to the activation of L-homoserine degradation pathway, we carried out a transcriptome analysis of the four strains (strains MG1655 or WT, 4E, MG1655thrL* and MG16555*ΔthrL*). While a complete transcriptomic analysis highlighted global gene expression changes in these strains versus WT is reported elsewhere (Alkim et al., *Data in Brief*, under submission), we focused the comparative transcriptomic analysis on genes that belong to superpathways of aspartate, L-threonine and glycine as they are connected to L-homoserine metabolism. As shown in Figure 6, genes of the *thrABC* operon were strongly upregulated in strains 4E and MG1655 thrL* as compared to WT. However, only in strain 4E was found a potent upregulation of *tdh* and *kbl* that belong to the threonine degradation pathway II (TDGII; see https://biocyc.org/META/new-image?object=THREONINE-DEG2-PWY) and of genes of *gcvTHP* operon that encodes the glycine cleavage system (GCS). In addition, expression of *alaC* encoding one of the three alanine: pyruvate transaminase was also increased by 15 fold, whereas *alaA* and *avtA* encoding the two other alanine transaminase were downregulated. Upon incubation with L-homoserine, genes of the GCS were upregulated in MG1655thrL* and slightly further activated in strain 4E, while those of the *thrABC* operon was not further increased. By contrast, incubation of WT and *ΔthrL* mutant with L-homoserine caused a transcriptional activation of the TDGII and GCS pathway (Figure 6B), but surprisingly, genes of the *thrABC* operon were downregulated. Altogether, these transcriptomic data are to a large extent consistent with the exometabolome data. Furthermore, these data showed the critical importance of the genomic modification at *thrL* locus leading to a thrL* allele allowing specific activation of the *thrABC* operon essential to mitigate L-homoserine toxicity.

### Growth with L-homoserine as the sole nitrogen source in strain 4E due to activation of TDGII and GCS

**Figure 7:**
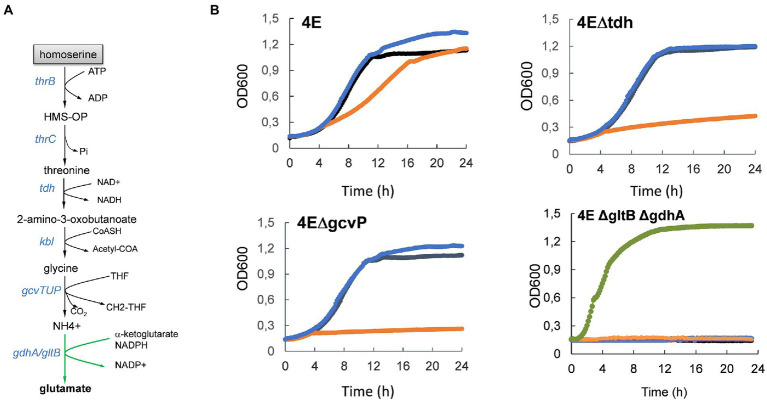
Activation of threonine degradation pathway II enables growth on L-homoserine as sole nitrogen source. In **(A)** is illustrated the metabolic route of L-homoserine degradation *via* threonine degradation pathway II that encompasses threonine dehydrogenase and 2-amino-3 oxobutanoate CoA ligase encoded by *tdh* and *kbl* respectively, followed by the glycine cleavage system encoded by *gcvTHP* operon. In **(B)** is shown the effect of loss of the threonine degradation pathway II or of GCS on growth in M9 with NH_4_^+^, NH_4_^+^ and L-homoserine or L-homoserine alone as nitrogen source. The M9 medium buffered at pH 7.0 with 100 mM MOPS contained 2% (w/v) glucose and growth was carried at 30°C in a Biotek. The black line is the growth medium with NH_4_CL (2 g/l) as nitrogen source, the blue line is growth medium M9 with NH_4_CL + 15 mM L-homoserine, orange line is the growth medium with 15 mM L-homoserine and the green line is growth on LB medium. Data are the mean of three technical replicates. 4E = L-homoserine-evolved strain; 4E*Δtdh* = −L-homoserine evolved strain deleted of tdh, 4E*Δkbl* = L-homoserine evolved strain deleted of *kbl*, 4E*ΔgcvP* = L-homoserine evolved strain deleted of *gcvP*.

The finding that *tdh* and *kbl* encoding enzymes of the threonine degradation pathway II (TDGII) and genes of the *gcvTHP* glycine cleavage system (GCS) (Table 3 and Figure 7A) were impressively upregulated only in the strain 4E prompted us to investigate whether the transcriptional activation of these genes was implicated in the ability of this evolved strain to grow with L-homoserine as nitrogen source. Figure 7B clearly showed that abrogation of TGDII or GCS by deletion of *tdh* or *gcvP* resulted in the complete inability of strain 4E to grow on M9 with L-homoserine as the nitrogen source. Nonetheless, the loss of function of the TDGII or GCS pathway did not interfere with the alleviation of strain 4E to L-homoserine toxicity in an ammonium-based M9 medium. Since the degradation of L-homoserine by the TDGII and GCS pathways ended up with CO_2_ and NH_4_^+^ (Figure 7A), it is very likely that the ammonium ions released in this pathway could be recaptured by glutamate dehydrogenase encoded by *gdhA* and *gltB*, allowing growth on L-homoserine. However, this could not be directly validated since the loss of function of this pathway predictably prevented growth on mineral M9 medium but not on LB medium, which is known to contain a plethora of amino acids (Figure 7B).

**Table 3:** The transcription factor encoded by *nac* potentially responsible for transcriptional activation of genes of the TDGII and GCS pathway in response to L-homoserine.

**Gene name**		**Product**	Strains*			**4E versus WT**	**WT_HMS *vs* WT**	**ΔthrL_HMS *vs* ΔthrL**
*tdh*	threonine dehydrogenase	78,20	3,73	3,71
*kbl*	2-amino-3-ketobutyrate CoA ligase	63,41	2,65	3,18
*nac*	nitrogen assimilation control protein	20,10	9,63	1,53
*gcvT*	aminomethyltransferase	15,66	3,97	2,24
*gcvH*	glycine cleavage complex, carrier of aminomethyl moiety	15,32	3,67	3,58
*gcvP*	glycine decarboxylase, P protein of glycine cleavage system	4,54	2,89	3,60

*Expression of genes in strains cultivated in the presence versus in the absence of L-homoserine, except for L-homoserine –evolved 4E strain in which the transcriptional comparison is made with the non-evolved MG1655 (WT). Values are fold change from three replicates at *p* value <0.01.

The next question was to understand how the genes of the TDGII and GCS pathways were upregulated in strain 4E. As noted above, the expression of these genes was also increased in the WT and *ΔthrL* strains upon incubation with L-homoserine, albeit to a lesser extent, indicating that this transcriptional effect is related to the presence of this amino acid. Also, it is worth noting that these genes harbor in the promoter region a binding site for DNA-binding transcriptional regulator encoded by *nac* (see Supplementary Figure S7 and https://ecocyc.org/gene?orgid=ECOLI&id=G7072#tab=REGULON) reported to regulate expression of genes involved in nitrogen metabolism under nitrogen-limiting / conditions (Muse and Bender, 1998), which can be mimicked by L-homoserine. Intriguingly, expression of *nac* expression was also strongly increased (20 fold) in strain 4E, and in WT (9 fold) but not in *ΔthrL* mutant upon incubation with L-homoserine (Table 3). However, loss of *nac* function did not prevent growth of strain 4E on a M9 medium with L-homoserine (data not shown), suggesting that transcriptional activation of genes of the TDGII and GCS pathways are not dependent on this transcription factor.

### Molecular targets of L-homoserine inferred by transcriptomic analysis

**Figure 8:**
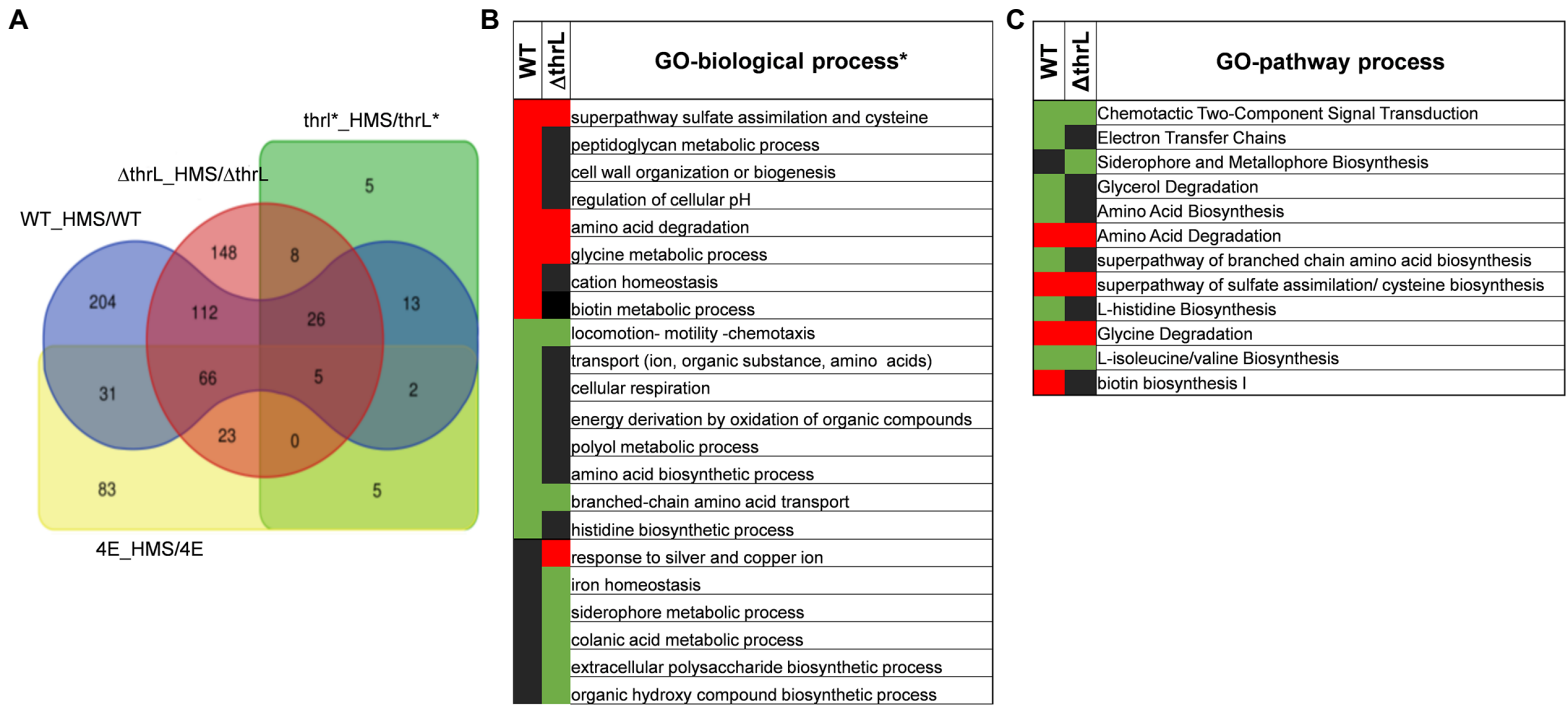
Effect of L-homoserine on the transcriptome of the MG1655 (WT), MG1655 carrying the allele (thrL*) or deleted for *thrL* (*ΔthrL*) and homoserine-adapted HMSCR4E (4E). The Venn diagram **(A)** shows the overlap of differentially expressed genes between the strains treated with L-homoserine versus untreated compared to the wild type strain. In **(B,C)** are reported genes ontology analysis in terms of biological **(B)** and metabolic processes **(C)** that are the most affected in the transcriptomes of WT and *ΔthrL* mutant in response to L-homoserine versus untreated. The concentration of L-homoserine used in the culture medium was 10 mM.

While ALE is a powerful strategy for unlocking certain nutritional restrictions or reducing toxicity to certain molecules, as we reported here for L-homoserine, this approach, however, provided little information about the molecular targets of L-homoserine toxicity. Therefore, to infer on these potential targets, genome-scale transcriptome analysis was carried out with the four strains (ie MG1655/WT, MG1655*ΔthrL*, MG1655thrL* and 4E) cultivated in a M9 glucose/ammonium medium in the absence and in the presence of 10 mM L-homoserine (a complete description on the experimental design, quality control and data are reported in an accompanying paper under submission to *Data In Brief)*. As shown in Figure 8A, the presence of 10 mM L-homoserine in the culture medium triggered the expression changes of 482 genes (about 10% of total genes) in WT, which was distributed in 234 up-regulated and 249 down-regulated genes. An almost comparable number of differentially expressed genes (428 in total, 272 upregulated and 156 downregulated genes) was found in the *ΔthrL* mutant, whereas this number was reduced to 192 in 4E and dropped to only 54 genes in MG1655thrl*. The smaller effect of L-homoserine on the transcriptome of the two latter strains could be explained by the fact that this amino acid had been completely depleted by the time the samples for transcriptome analysis were taken, which was about 6 h after the start of the culture (see Figure 5). We therefore focused our analysis of the effects of L-homoserine on WT and *ΔthrL* mutant and performed gene ontology (GO) analysis using EcoCyc tools (see Footnote 5; Keseler et al., 2016) to highlight the most altered biological processes in response to L-homoserine. This analysis revealed biological processes/pathways that were commonly enriched in both strains, indicating that they were likely targets of L-homoserine and also differences between the two strains, revealing specific effects linked to *thrL* gene.

**Figure 9:**
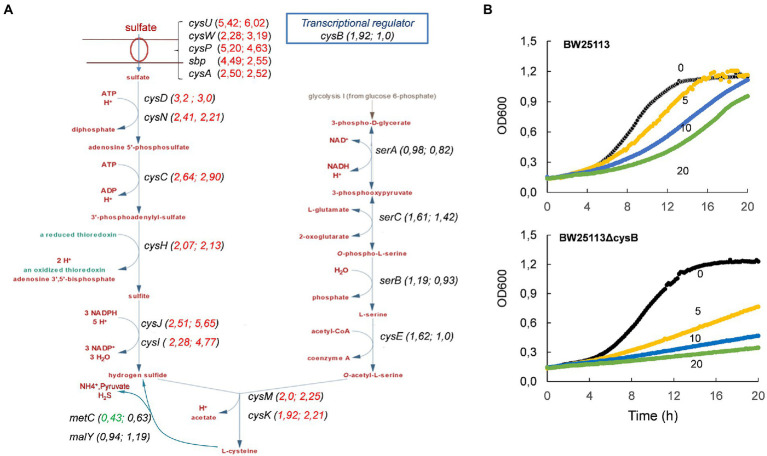
Genes encoding sulfate uptake and assimilation are upregulated in MG1655 (WT) and MG1655*ΔthrL* strains during growth in M9 medium buffered at pH 7.0 with 100 mM MOPS contained 2% (w/v) glucose in the presence of 10 mM L-homoserine. In **(A)** is shown the change in gene expression (in parentheses) in the metabolic map, starting with sulfate transport to hydrogen sulphide condensation with O-acetyl serine to produce cysteine. The first value in parentheses corresponds to that of WT and the second to *ΔthrL* mutant strain. In **(B)** is shown that the loss of *cysB* in strain BW25113 enhance its sensitivity to L-homoserine. Concentration of L-homoserine added to the medium is reported on the growth curves.

Both WT and *ΔthrL* strains had in common a striking down-regulation of genes that belong to the locomotion-motility-chemotaxis process and to metabolism and transport of branched chain amino acids (Figures 8B,C). Quantitatively, expression of genes in these biological processes were much repressed in the WT (up to 100 fold) than in the *Δthrl* mutant (e.g., 5 fold max) (see Tables 4, 5), suggesting that the effect of L-homoserine to repress these genes may depend in part on the function of *thrL*. The list of these repressed genes was then used to search for their cognate transcription factors using EcoCyc tool,^9^ which identified *flhC* and *flhD* encoding DNA-binding transcriptional activator of flagellar genes (*p* value <10^−27^) that are implicated in bacterial motility (Prüß, 2017). These transcriptional factors could be therefore a target of L-homoserine. The repression of genes encoding branched chain amino acids LIV-I and LS system transporters, which was also shown to import L-homoserine (Adams et al., 1990) was consistent with the finding that the loss of these transport systems did not alter the toxicity of this amino acid. Also, common to WT and *ΔthrL* strains was the upregulation of all genes (ranging from 2.5 to 6 fold) that belong to the uptake and reductive assimilation of sulfate until cysteine (Figure 9). Since the activity of this pathway is energetically costly (3 NADPH, 2 ATP and the energetic cost of sulfate import), it can be assumed that the growth-restrictive effects of L-homoserine is a consequence of this high energy cost. However, proof of this suggestion should require a metabolic flux analysis using ^35^-SO_4_ for instance. It is also worth noticing that the genes of this pathway are under the control of the transcriptional factor encoded by *cysB*, and that this activation requires the binding of N-acetyl serine (NAS) to CysB (Ostrowski and Kredich, 1989; Ostrowski and Kredich, 1990). Therefore, assuming that the effects of L-homoserine in activating these genes are mediated by CysB, the gene encoding this factor was deleted and contrary to expectation, the *ΔcysB* mutant turned out to be even more sensitive to L-homoserine than the wild type (Figure 9B).

**Table 4:** List of genes that belong to the locomotion-chemotaxis-motility process that were downregulated in WT and *ΔthrL* mutant in response to L-homoserine.

**Gene name**	**Product**	**WT_HMS**	**Δthrl_HMS**
*cheW*	positive regulator of CheA protein activity	0.009	0.203
*flgE*	flagellar biosynthesis, hook protein	0.009	0.183
*fliC*	flagellar biosynthesis	0.009	0.172
*flgD*	flagellar biosynthesis, initiation of hook assembly	0.010	0.178
*flgG*	flagellar biosynthesis, cell-distal portion of basal-body rod	0.010	0.146
*flgB*	flagellar biosynthesis, cell-proximal portion of basal-body rod	0.011	0.151
*flgC*	flagellar biosynthesis, cell-proximal portion of basal-body rod	0.011	0.204
*flgF*	flagellar biosynthesis, cell-proximal portion of basal-body rod	0.013	0.157
*cheY*	chemotaxis regulator to flagelllar motor components	0.013	0.251
*motA*	proton conductor component of motor	0.016	0.345
*flgK*	flagellar biosynthesis, hook-filament junction protein 1	0.016	0.252
*fliM*	flagellar biosynthesis, component of motor switch	0.016	0.238
*cheA*	sensory transducer kinase	0.019	0.286
*fliD*	flagellar biosynthesis	0.020	0.366
*flgA*	flagellar biosynthesis	0.023	0.203
*tar*	methyl-accepting chemotaxis protein II, aspartate sensor receptor	0.023	0.381
*cheR*	response regulator for chemotaxis	0.025	0.357
*fliS*	flagellar biosynthesis	0.025	0.338
*flgL*	flagellar biosynthesis	0.026	0.255
*motB*	enables flagellar motor rotation, linking torque machinery to cell wall	0.027	0.366
*fliF*	flagellar biosynthesis	0.027	0.309
*tsr*	methyl-accepting chemotaxis protein I, serine sensor receptor	0.028	0.535
*cheB*	response regulator for chemotaxis	0.029	0.413
*fliL*	flagellar biosynthesis	0.033	0.213
*fliJ*	flagellar fliJ protein	0.035	0.189
*flgH*	flagellar biosynthesis, basal-body outer-membrane L	0.036	0.218
*tap*	methyl-accepting chemotaxis protein IV, peptide sensor receptor	0.040	0.468
*fliN*	flagellar biosynthesis, component of motor switch and energizing,	0.043	0.233
*fliH*	flagellar biosynthesis	0.043	0.325
*fliT*	flagellar biosynthesis	0.049	0.550
*fliG*	flagellar biosynthesis,	0.051	0.297
*flgI*	homolog of Salmonella P-ring of flagella basal body	0.055	0.262
*flgJ*	flagellar biosynthesis	0.060	0.365
*fliK*	flagellar hook-length control protein	0.060	0.299
*fliI*	flagellum-specific ATP synthase	0.063	0.351
*cheZ*	chemotactic response	0.092	0.610
*fliO*	flagellar biosynthesis	0.138	0.327
*fliE*	flagellar biosynthesis	0.139	0.702
*aer*	aerotaxis sensor receptor, flavoprotein	0.162	0.718
*trg*	methyl-accepting chemotaxis protein III, ribose sensor receptor	0.283	0.930

Values given are fold change from 3 replicates at *p* value <0.01.

**Table 5:** Genes encoding component of the branched amino acid transport system downregulated in WT and *ΔthrL* strains in response to L-homoserine.

**Gene name**	**Product**	**Fold change WT_HMS**	**Fold change Δthrl_HMS**
*livF*	ATP-binding component of leucine transport	0.083	0.118
*livH*	high-affinity branched-chain amino acid transport system	0.088	0.116
*livM*	high-affinity branched-chain amino acid transport	0.095	0.106
*livK*	high-affinity leucine-specific transport system	0.051	0.095
*livG*	ATP-binding component of high-affinity branched-chain amino acid transport system	0.072	0.089
*livJ*	high-affinity amino acid transport system	0.013	0.025

This transcriptomic analysis also revealed that biological processes that were up-regulated in the WT grown in the presence of L-homoserine (e.g., cell wall/peptidoglycan metabolic process and biotin biosynthesis) or down-regulated (e.g., cell respiration, histidine biosynthesis) were not observed in the *thrL*-defective mutant (Figures 8B,C), suggesting that these L-homoserine-mediated transcriptomic changes require the presence of a functional *thrL*. Conversely, a down-regulation of colonic acid and extracellular polysaccharide biosynthesis and a potent upregulation of genes implicated in the response to silver and copper were observed specifically in *ΔthrL* mutant cultivated with L-homoserine (Figure 8B; Table 6). However, alteration of these biological processes already occurred upon deletion of *thrL* but very surprisingly, with the opposite effect (Table 6). These data were rather puzzling but may suggest that L-homoserine cancelled the effects of *thrL* deletion on the expression of these genes by returning them to an expression level comparable to that in WT, as suggested by the calculated expression values obtained by multiplying the fold change of *ΔthrL*/WT with that of *Δthrl*-HMS/*ΔthrL* (Table 6).

**Table 6:** Genes whose expression is increased or decreased in *ΔthrL* mutant versus WT while the opposite change occurred when the *Δthrl* mutant is treated with 10 mM L-homoserine.

	**Gene name**	**Product**	*Fold change *ΔthrL*/wt	*Fold change *ΔthrL*_HMS/*ΔthrL*	**Calculated Fold change (ΔthrL/WT) X (ΔthrL_HMS/DthrL)**
**colanic and extracellular polysaccharide metabolic process**	*cpsB*	mannose-1-phosphate guanyltransferase	4.95	0.21	1.03
	*cpsG*	phosphomannomutase	2.59	0.32	0.83
	*fcl*	putative nucleotide di-P-sugar epimerase or dehydratase	3.92	0.25	0.98
	*gmd*	GDP-D-mannose dehydratase	4.91	0.18	0.88
	*gmm*	GDP-mannose mannosyl hydrolase	3.23	0.35	1.13
	*wcaB*	colanic acid biosynthesis acetyltransferase	4.42	0.25	1.10
	*wcaC*	colanic acid biosynthesis galactosyltransferase	3.82	0.21	0.67
	*wcaD*	putative colanic acid polymerase	4.86	0.21	1.02
	*wcaE*	GDP-Fuc:2/3-*O*-Ac-α-L-Fuc-(1 → 3)-α-D-Glc-PP-Und α-(1,4)-fucosyltransferase	4.46	0.22	0.98
	*wcaF*	colanic acid biosynthesis acetyltransferase	2.62	0.39	1.02
	*wcaI*	colanic acid biosynthesis fucosyltransferase	2.19	0.35	0.77
	*wza*	outer membrane polysaccharide export protein	4.43	0.23	1.02
	*wzb*	protein-tyrosine phosphatase	4.82	0.21	1.01
	*wzc*	protein-tyrosine kinase	4.16	0.25	1.04
	*wzxC*	colanic acid repeat unit flippase	3.04	0.35	1.19
	*yjbE*	orf, hypothetical protein	6.65	0.18	1.2
	*yjbF*	orf, hypothetical protein	2,30	0.38	0.87
	*yjbG*	orf, hypothetical protein	2.09	0.47	0.98
**response to silver and copper ions**	*cusC*	copper/silver export system outer membrane channel	0.005	229,34	1.14
	*cusF*	copper/silver export system periplasmic binding protein	0.009	62,17	0.55
	*cusB*	copper/silver export system membrane fusion protein	0.019	30.57	0.58
	*copA*	soluble Cu^+^ chaperone	0.048	10.95	0.53
	*cueO*	multicopper oxidase	0.057	6.86	0.40
	*cusA*	copper/silver export system RND permease	0.072	18,94	1.36
	*cusR*	DNA-binding transcriptional activator	0.085	6.22	0.52
	*cusS*	sensor histidine kinase	0.087	11.36	0.99

*Value are the fold change from 3 replicates at *p* value <0.01.

## Discussion

In this work, we aimed to explain why *E. coli* is unable to use L-homoserine as a nitrogen source and whether this inability is related to the toxic effect of this non-canonical amino acid on growth of this bacteria. To this end, we employed an adapted laboratory evolution (ALE) strategy that allowed the isolation of an evolved strain 4E capable of growing on the mineral medium M9 with L-homoserine as sole nitrogen source. Interestingly, the growth of this adapted strain on a conventional mineral medium was no longer inhibited by L-homoserine, which may support the view that these two L-homoserine features were independent. On the one hand, the alleviation of L-homoserine toxicity was due to activation of a detoxification process that readily converts this molecule into the nontoxic amino acid threonine. This detoxification corresponded to a potent transcriptional activation of the *thrABC* operon genes that was strictly associated with a genomic change at the *thrL* locus and involved a 49-bp truncation starting at the stop codon of *thrL* and extending through the Rho-independent terminator sequence of the natural gene. This truncation led to a new genomic locus carrying a thrL* allele encoding a polypeptide 9 amino acids longer than the wild-type ThrL polypeptide [30 vs. 21 amino acids in length (Lynn et al., 1982, 1987)] that was entirely responsible for the activation of the *thrABC* operon but did not allow growth on a medium with L-homoserine as nitrogen source. On the other hand, the ability of *E. coli* to grow on a mineral medium with L-homoserine was strictly dependent on the threonine degradation pathway II (TDGII) and the glycine cleavage system (GCS), through transcriptional activation of their corresponding genes (*tdh*, *kbl* and *gcvTHP*, respectively). Thus, the growth on L-homoserine can be explained by the complete degradation of this amino acid, which ends up with CO_2_ and ammonium ions. These latter served as direct nitrogen source for the cell, being recaptured by the *gdhA* and *gltB* –encoded glutamate dehydrogenase. However, it is not known how the genes of the TDGII and GCS pathways were transcriptionally activated in strain 4E, because the increase in their expression was not associated with either the genomic mutations identified in this strain or with transcriptional activation of *nac*, which encodes a transcription factor involved in adaptation to nitrogen-limiting conditions (Muse and Bender, 1998; Switzer et al., 2018), even though these genes have a binding site for this TF in their promoter. On the other hand, the transcriptional activation of these genes were clearly specific to the presence of L-homoserine since they were also upregulated in WT and *ΔthrL* mutant incubated with this compound, albeit to a lower extent.

Although ALE is a powerful enabling technology for adapting microbial cells to toxic compounds, it may not easily decipher the molecular mechanism underlying the toxicity because very often it activates a detoxification process leading to rapid metabolization of the toxic compound into a less or nontoxic molecule, as it was reported for furfural, acetic acid, or phenolic toxic compounds that inhibit yeast fermentation (Lewis et al., 2009; Koppram et al., 2012; Jung et al., 2017). Therefore, to get some clues on the mechanism of L-homoserine toxicity in *E. coli,* we turned out to a transcriptomic analysis which revealed that nearly 10% of the *E. coli* genome was transcriptionally modified in the presence of 10 mM L-homoserine in the growth medium. The locomotion - motility - chemotaxis process was found to be strongly repressed with genes downregulated more than 100 fold in response to L-homoserine. The repression of this biological process can be part of the growth inhibition effect exerted by L-homoserine, since a deficiency in locomotion-motility-chemotaxis has been reported to be associated with a reduction in growth rate (Sim et al., 2017). This growth inhibition is likely not due to N-acyl-homoserine lactone (AHL) dependent quorum sensing since *E. coli* does not contain the AHL synthase encoding gene (Van Houdt et al., 2006). In addition, the finding that the repression of this biological process was weaker in a *ΔthrL* mutant goes along with the fact that the sensitivity of this mutant strain to L-homoserine was about 2.5 times lower than that of the WT. Whether the repression of genes that belong to this process is mediated through inhibition of the transcription activators of flagellar genes encoded by *flhC* and *flhD* (Bartlett et al., 1988) by L-homoserine is an open question. The upregulation of all genes implicated in the reductive assimilation of sulfate is clearly another target of L-homoserine. In addition to the fact that this metabolic pathway is energy and redox intensive, the fact that deletion of *cysB* encoding the transcriptional activator of genes in this pathway (Kredich, 1992), makes *E. coli* even more sensitive to inhibition by L-homoserine was surprising. Although there is no trivial explanation for this result, it clearly indicates that the effects of L-homoserine on these genes are independent of those of *cysB*. Finally, our transcriptomic analysis revealed a still unexplained behavior of L-homoserine to antagonize transcriptional changes caused by *thrL* deletion, which are a repression of genes involved in the biosynthesis of colanic acid and extracellular polysaccharides and activation of genes of the response to copper/silver ion detoxification system. How the peptide leader ThrL may affect these processes remains to be studied.

## Conclusion

In this work, an *in vivo* evolutionary engineering strategy was used to isolate an *E. coli* strain able to grow on a medium with L-homoserine as the nitrogen source, which at the same time became insensitive to growth toxicity by this non-canonical amino acid. This strategy was very successful as it showed that these two phenotypic traits brought about by L-homoserine were functionally distinct. On the one hand, alleviation of L-homoserine toxicity resulted from activation of a detoxification process that rapidly converts L-homoserine to threonine. This process was found to be dependent on the expression of a mutated thrL* gene that resulted from a 49 bp truncation starting at the stop codon sequence of *thrL*. Remarkably, this genomic modification transformed the function of ThrL from an attenuator to a transcriptional activator of the threonine synthesis *thrABC* operon. On the other hand, the ability to grow on L-homoserine was due to the activation of the TDGII and GCS pathways resulting from the transcriptional activation of their corresponding genes. Transcriptomic analyses of L-homoserine-treated WT allowed us to deduce possible molecular targets that may account for the homoserine toxicity. In particular, genes implicated in locomotion-motility-chemotaxis process were severely repressed while all genes encoding the reductive assimilation of sulfate, including their transporters, were strongly activated in cells incubated with L-homoserine.

## Data availability statement

The datasets presented in this study can be found in online repositories. The names of the repository/repositories and accession number(s) can be found at: https://www.ncbi.nlm.nih.gov/geo/, GSE206196.

## Author contributions

CA and JMF designed the experiments and wrote the manuscript, which was improved and approved by all other authors. CA, DF, JF, HS-B, and NS carried out the molecular experiments. Adaptive laboratory evolution using GM3 automate was carried out by MP. CA, DF, and PH carried out the physiological and metabolomics analysis. SD helped in performing the statistical and biomathematical data analysis. NM and BE contributed to metabolic and transcriptomic data analyses. Funding acquisition and supervision were carried out by JMF. All authors contributed to the article and approved the submitted version.

## Funding

This work was supported by Grant no. 1782C0056 from ADEME (Agence de l’environnement et de la maîtrise de l’Energie - Plan Investissement Avenir 2) and no. BTBR05-01 from ANR (Agence National de la Recherche - Plan Investissement Avenir 1).

## Conflict of interest

The authors declare that the research was conducted in the absence of any commercial or financial relationships that could be construed as a potential conflict of interest.

## Publisher’s note

All claims expressed in this article are solely those of the authors and do not necessarily represent those of their affiliated organizations, or those of the publisher, the editors and the reviewers. Any product that may be evaluated in this article, or claim that may be made by its manufacturer, is not guaranteed or endorsed by the publisher.

## Footnotes

^1^ https://www.labsexplorer.com/lab/heurisko-by-altar_104976

^2^ https://www.atum.bio/eCommerce/cas9/input

^3^ https://get.genotoul.fr/en/

^4^ http://mixomics.org/

^5^ https://ecocyc.org/

^6^ https://ecocyc.org/groups?orgid=ECOLI
