# Supplementary material for: Toxic effect and inability of L-homoserine to be a nitrogen source for growth of Escherichia coli resolved by a combination of in vivo evolution engineering and omics analyses
Source: Front Microbiol. 2022 Dec 13;13:1051425. doi: 10.3389/fmicb.2022.1051425 (PMC9792984; doi:10.3389/fmicb.2022.1051425)
Supplement: Supplementary file 1 [file Data_Sheet_1.PDF]

Supplementary data

Toxic effect and inability of L-homoserine to be a nitrogen source for growth of *Escherichia coli* resolved by a combination of i*n vivo* evolution engineering and omics analysis.

Ceren Alkim[1,2], Daniele Farias[1], Julie Fredonnet[2], Helene Serrano-Bataille[2], Pauline Herviou[2], Marc Picot[1], Nawel Slama[2], Sebastien Dejean[3], Nicolas Morin[2], Brice Enjalbert[1] and Jean M François[1,2*]

[1]Toulouse Biotechnology Institute (TBI), Université de Toulouse, CNRS, INRA, INSA, Toulouse, France, 135 Avenue de Rangueil, F-31077 Toulouse, France;

[2]Toulouse White Biotechnology center (TWB), UMS-INSA-INRA-CNRS, 135 avenue de Rangueil, F-31077 Toulouse

[3]Institut de Mathématique Toulouse, 135 avenue de Rangueil, 31077 Toulouse, cedex 04

Key words:

Escherichia coli, Microbial physiology, L-homoserine, genetic regulation, transcriptomics, evolutionary engineering

Email address: alkim@insa-toulouse.fr
julie.fredonnet@inrae.fr
helene.serrano@micro-pep.com
daniele.farias@insa-toulouse.fr
pauline.herviou@inrae.fr
marc.picot@inrae.fr
nawel.slama@inrae.fr
sebastien.dejean@math.univ-toulouse.fr
nicolas.morin@inrae.fr
brice.enjalbert@insa-toulouse.fr
fran_jm@insa-toulouse.fr or jean.marie-francois@inrae.fr

*Address for correspondence: fran_jm@insa-toulouse.fr

Table S1: List of *E. coli* Strain and plasmids used in this study

Strain	Genotype	Source
MG1655	K12 $F^-\lambda^-$ *ilvG*$^-$ *rfb-50 rph-1*	ATCC 47076
BL21(DE3)	fhuA2 [lon] ompT gal (λ DE3) [dcm] ΔhsdS λ DE3 = λ sBamHIo ΔEcoRI-B int::(lacI::PlacUV5::T7 gene1) i21 Δnin5	New England Biolabs
Stellar	F–, endA1, supE44, thi-1, recA1, relA1, gyrA96, phoA, Φ80d lacZΔ M15, Δ (lacZYA - argF) U169, Δ (mrr - hsdRMS - mcrBC), ΔmcrA, λ–	New England Biolabs
NEB 5-alpha	fhuA2 Δ(argF-lacZ)U169 phoA glnV44 Φ80Δ (lacZ)M15 gyrA96 recA1 relA1 endA1 thi-1 hsdR17	New England Biolabs
DH10B	Δ(ara-leu) 7697 araD139 fhuA ΔlacX74 galK16 galE15 e14- φ80dlacZΔM15 recA1 relA1 endA1 nupG rpsL (StrR) rph spoT1 Δ (mrr-hsdRMS-mcrBC)	New England Bioloabs
NiCo21(DE3)	can::CBD fhuA2 [lon] ompT gal (λ DE3) [dcm] arnA::CBD slyD::CBD glmS6Ala ΔhsdS λ DE3 = λ sBamHIo ΔEcoRI-B int::(lacI::PlacUV5::T7 gene1) i21 Δnin5	New England Biolabs
BW25113	Δ(araD-araB) 567 Δ(rhaD-rhaB) 568 ΔlacZ4787 (::rrnB-3) hsdR514 rph-1	CGSC collection
JS200	SC-18 recA718 polA12 uvrA155 trpE65 lon-11 sulA1	{Camps, 2003 #10838}
MG1655 *ΔthrL*	MG1655 deleted of *thrL* by phage transduction	This work
4E	Evolved MG1655 on L-homoserine	This work
4E *ΔthrB*	4E deleted of *thrB* by phage transduction	This work
4E *Δtdh*	4E deleted of *tdhL* by phage transduction	This work
4E *Δkbl*	4Edeleted of *kbl* by phage transduction	This work
4E *ΔgcvP*	4E deleted of *gcvP* by phage transduction	This work
4E *Δgdha ΔgltB*	4E deleted of *gdhA gltB* by phage transduction	This work
4E*Δnac*	4E deleted of *nac* by phage transduction	This work
MG1655 *thrL**	MG1655 with replacement of wild type *thrL* by the truncated version *thrL** by CRISPR-cas9	This work
BW25113 *ΔlivJ*	F-, Δ(araD-araB)567, ΔlacZ4787(::rrnB-3), λ-, ΔlivJ790::kan, rph-1, Δ(rhaD-rhaB)568, hsdR514	{Baba, 2006 #9562}
BW25113 *ΔlivK*	F-, Δ(araD-araB)567, ΔlacZ4787(::rrnB-3), λ-, ΔlivK788::kan, rph-1, Δ(rhaD-rhaB)568, hsdR514	{Baba, 2006 #9562}
BW25113 *ΔtdcC*	F-, Δ(araD-araB)567, ΔlacZ4787(::rrnB-3), λ-, ΔtdcC732::kan, rph-1, Δ(rhaD-rhaB)568, hsdR514	{Baba, 2006 #9562}

Table S2: listing of plasmid used and constructed in this work

Plasmids	description	Source/reference
pET-28a(+)	Expression of different genes	Novagen
pET-28a alaC	pET-28-a(+) derivative carrying *E. coli alaC* gene	This study
pET-28a alaC^{R78G}	pET-28-a(+) derivative carrying *E. coli alaC*^{R78G} gene	This study
pCP20	plasmid used for removing Kan cassette	{Cherepanov, 1995 #9564}
pCas	Plasmid used for Cas9 and lambda RED, constitutive and inducible expression, respectively	{Jiang, 2015 #10453}
pTargetF	Plasmid that express sgRNA constitutively	{Jiang, 2015 #10453}
pTargetF-*thrL**	Plasmid used for targeting the region to be replaced by CRISPR-Cas9 method	This study

Table S3: listing of primers used in this work

oligonucleotide	description	Gene target
thrL-vrf-fw	Verification of *thrL* deletion	AACGGGCAATATGTCTCTGTG
thrL-vrf-rev		GATGTACCGCCGAACTTCAACA
thrL-seq-fw	To sequence *thrL-thrL** up and downstream regions	AGCTTTTCATTCTGACTGC
thrL-seq-rev		CAGAAAACGTTCTGCATTT
thrL*-BamHI-fw	To clone *thrL** into pET-28a(+)	ATTGTTGGATCCATGAAACGCATTAGCACC
thrL*-HindIII-rev		GTTCCAAAGCTTTTACCTCGTTACCTTTGG
thrL-donneur-fw	To amplify *thrL** donneur DNA	AGCTTTTCATTCTGACTGCAA
thrL-donneur-rev		CAAATTCCTGATCGACGAAAG
oligo-pTarget-thrL*	To construct pTargetF-*thrL**	GTCCTAGGTATAATACTAGT**CGCACCGTTACCTGTGGTAA**GTTTTAGAGCTAGAAATAGC*
pTargetF-rev		ACTAGTATTATACCTAGGACTGAG
gRNArvbis	To sequence pTargetF-thrL*	GTCGTTGATCAAAGCTCGCCGCGTTG
nac-vrf-fw	Verification of *nac* deletion	GGCAGTGCATGGTGATGTCAAAG
nac-vrf-rev		GTAATGCTTTGCCCGGTTCAGT
kbl-vrf-fw	Verification of *kbl* deletion	CAATCATCATTATGCCCAGCCA
kbl-vrf-rev		GCGGCAATGACCACAGGTGA
locus_tdh_FOR	Verification of *tdh* deletion	TATGCCAACAACGATATGCAGGAGC
locus_tdh_REV		TCATCGCCCGCATTATATAACGG
Ec_gltB_Fwd	Verification of *gltB* deletion	AACAAGGGGGCGAATGCGAG
Ec_gltB_Rev		TTGCTACCCCTTACTGCGCCTG
Ec_gdhA_Fwd	Verification of *gdhA* deletion	GCAAAAGCACATGACATAAACA
Ec_gdhA_Rev		GCCATCAGGCATTTACAACTTA

*N20 region is indicated by red color

Table S4: Kinetic properties of AlaC and AlaC^{R78G} variant*

Enzyme	Substrate	K_M (mM)	V^{max} (µmol/min/mg protein)	k_{cat} (s^{-1})	k_{cat}/K_M (s^{-1}. M^{-1})
**AlaC**	alanine	5.2	0.80	0.65	125
	α-ketoglutarate	0.09	0.65	0.53	5880
	pyruvate	0.10	0.64	0.52	5200
	glutamate	7.2	0.62	0.55	76
**AlaC^{R78G}**	alanine	2.8	1.1	0.92	328
	α-ketoglutarate	0.08	1.15	0.94	17638
	pyruvate	0.13	1.15	0.94	7230
	glutamate	2.20	1.20	1.06	482

*Data are the mean of two independent experiments

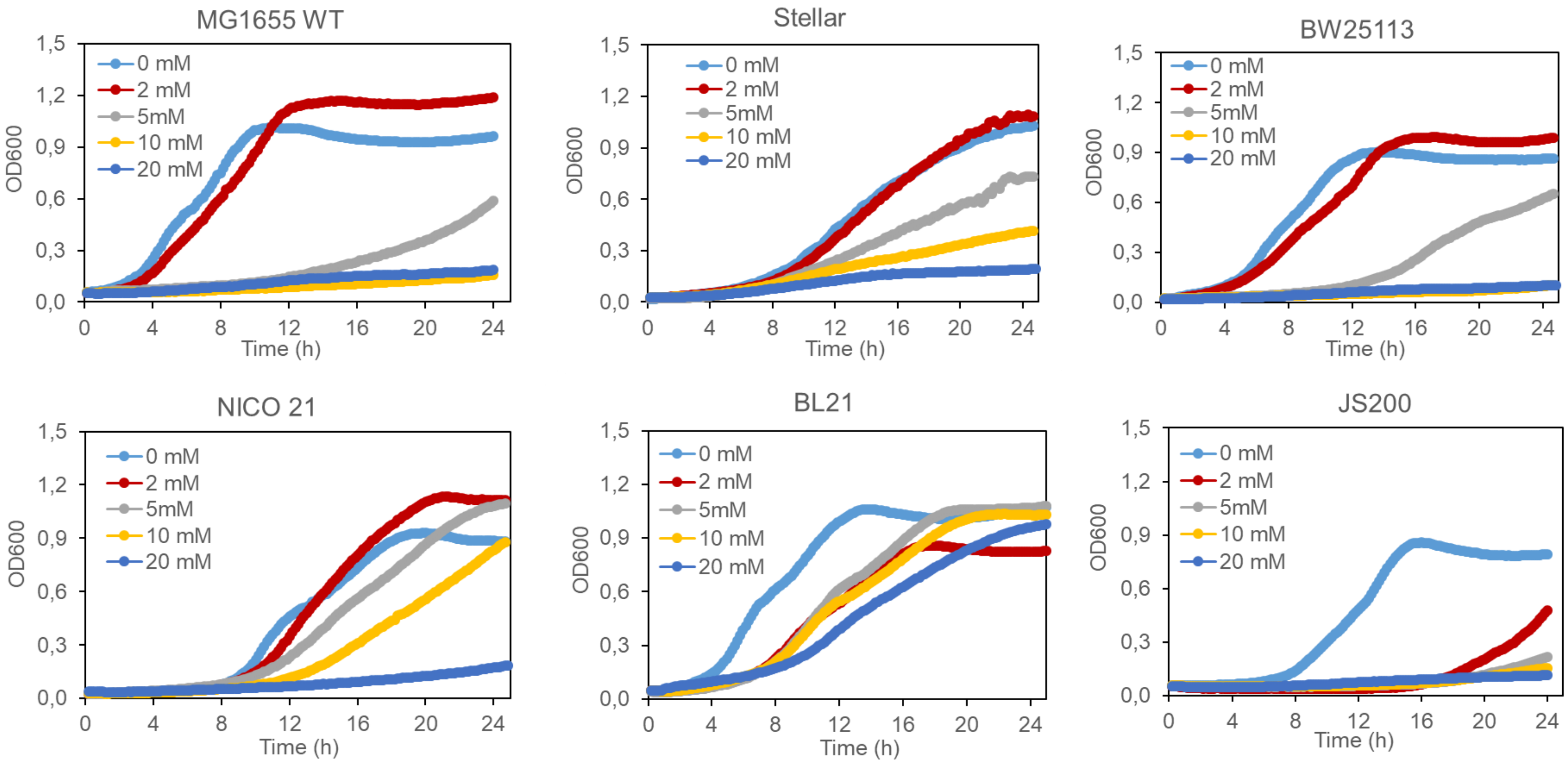

Figure S1: Homoserine causes growth inhibition when added to a mineral medium containing another source of nitrogen (ammonium ions). The growth was carried out in M9 medium buffered at pH 7.0 with 100 mM MOPS containing 0.4% (w/v) glucose at 30°C in a Biotek microplate reader.

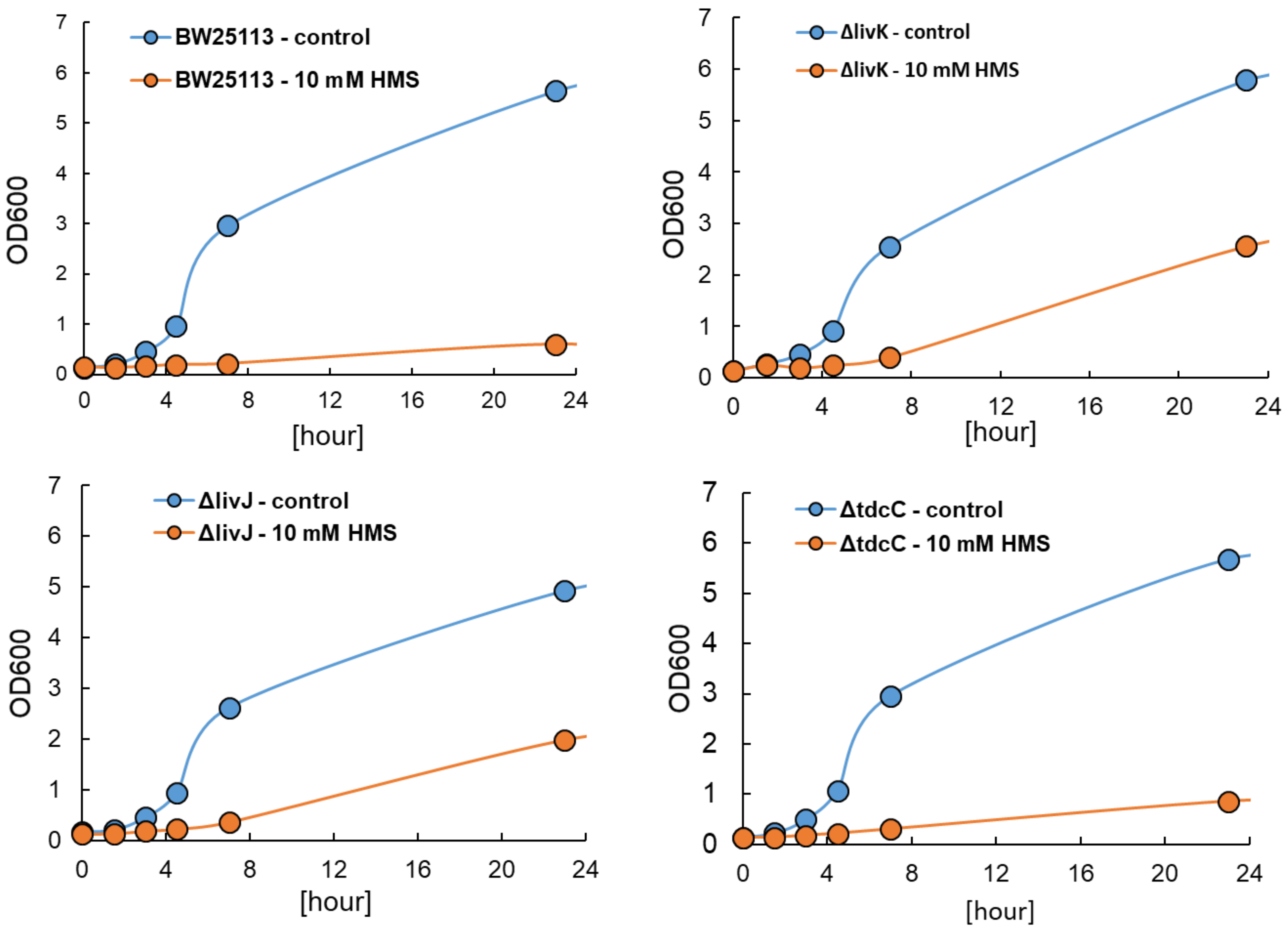

Figure S2. Deletion of *livJ or livK* encoding branched -chain amino acids transporters, or *tdcC* encoding the threonine importer does not abolish the growth inhibitory effect of homoserine. The growth was made in M9 buffered at pH 7.0 with 100 mM MOPS containining 2% (w/v) glucose

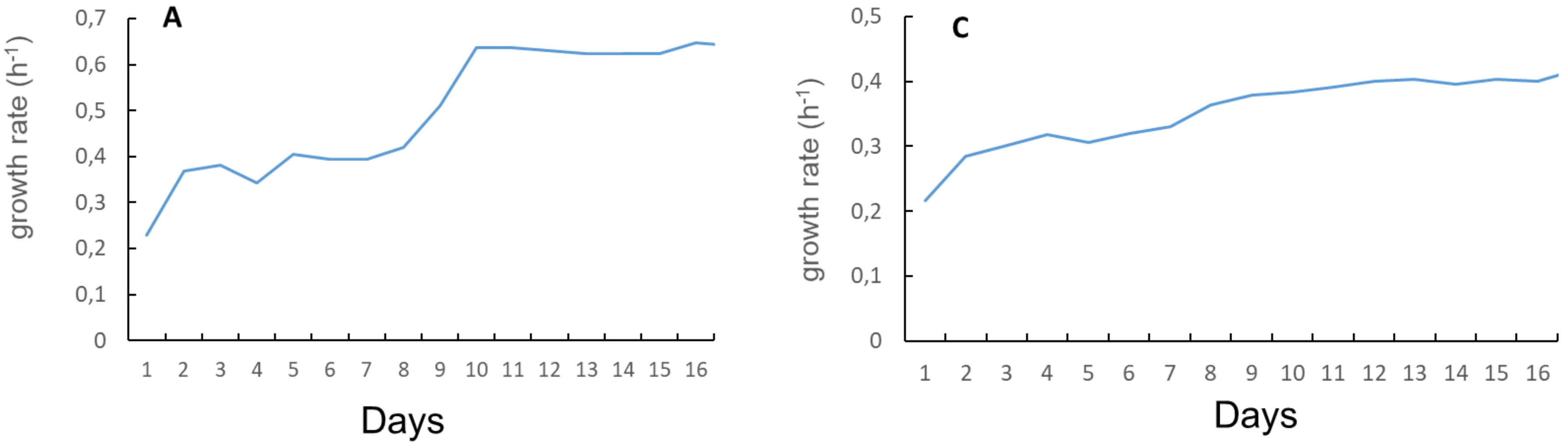

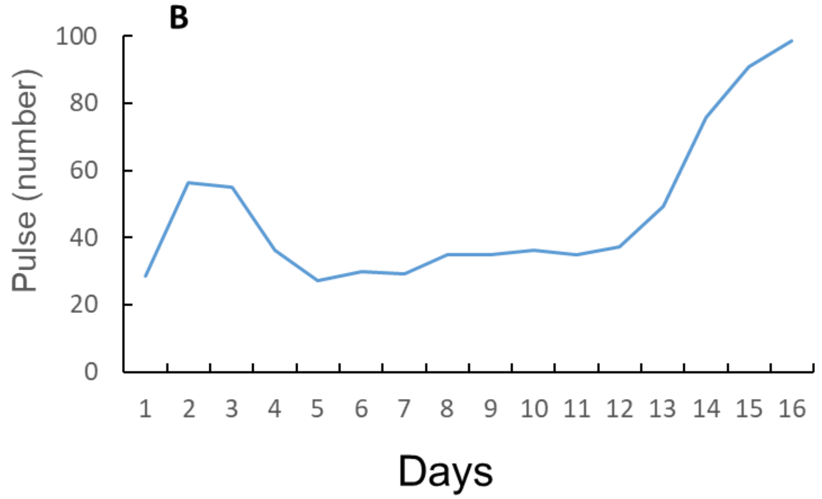

Figure S3: Homoserine-resistant WT MG1655 was obtained by evolutionary engineering. In (A), the culture was firstly adapted in a turbidostat mode to grow in M9 medium buffered at pH 7.0 with sodium phosphate/ citrate containing 0.2% (w/v glucose) and L-aspartate (10mM) as nitrogen sourec, until reaching the highest and stable growth rate. In (B), a medium swap mode was applied corresponding to pulse addition of a restrictive M9 medium with L-homoserine as the nitrogen source (10 mM) at constant a growth rate. Adaptation of the population on homoserine was obtained when % of pulse of restrictive medium reached 100%. In (C), the population was further adapted to L-homoserine restrictive medium using a turbidostat mode until it yielded a stable specific growth rate.

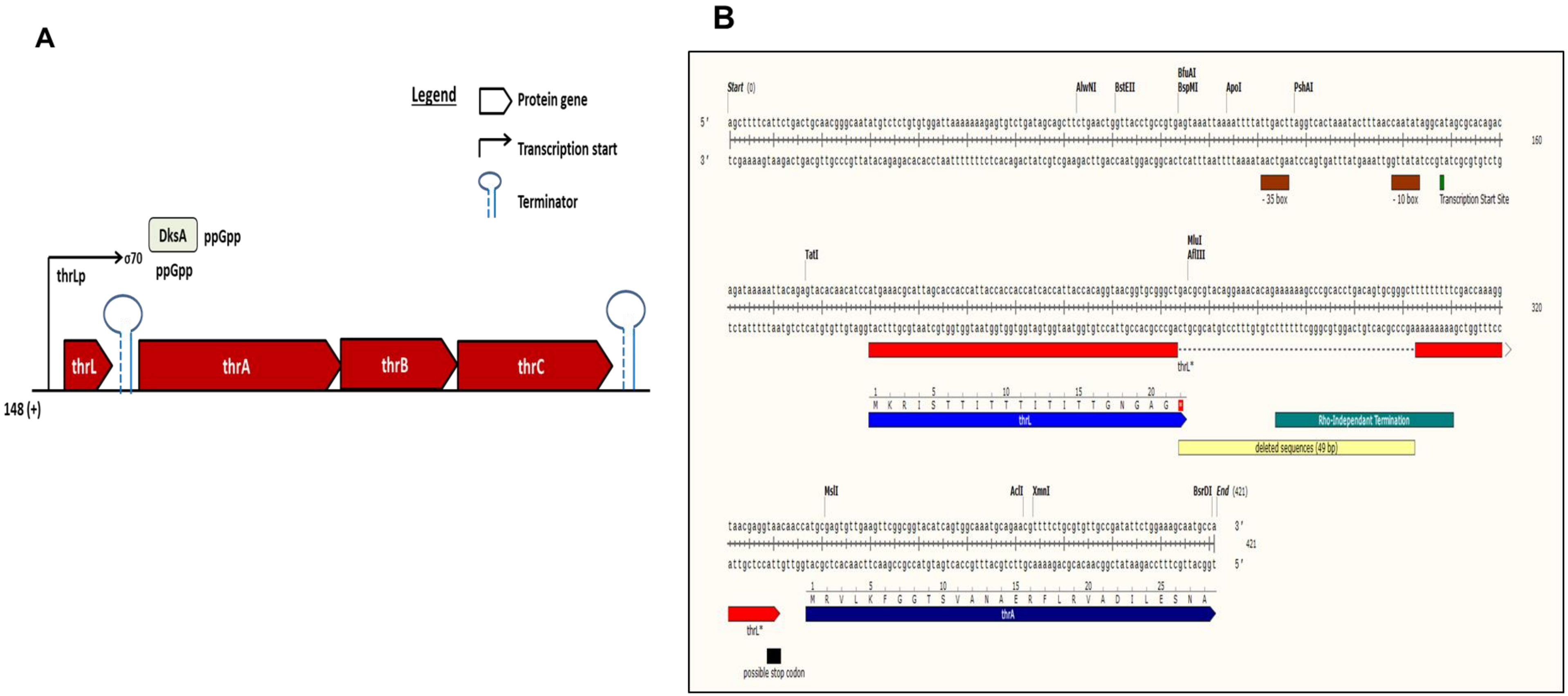

Figure S4: (A) *thrLABC* operon. Genes, terminator sites, transcription start site and transcription regulators of this operon were demonstrated. (Keseler et al. (2017), "EcoCyc: reflecting new knowledge about Escherichia coli K-12", Nucleic Acids Research 45:D543-50.). (B) sequencing data at the *thrL* locus of the wild type MG1655 and the HMSRCE4 strain. The blue bar shows the wild type *thrL* gene, while the red bar show the *thrL** allele in HMSRCE4, with the deleted region highlighted by the yellow bar.

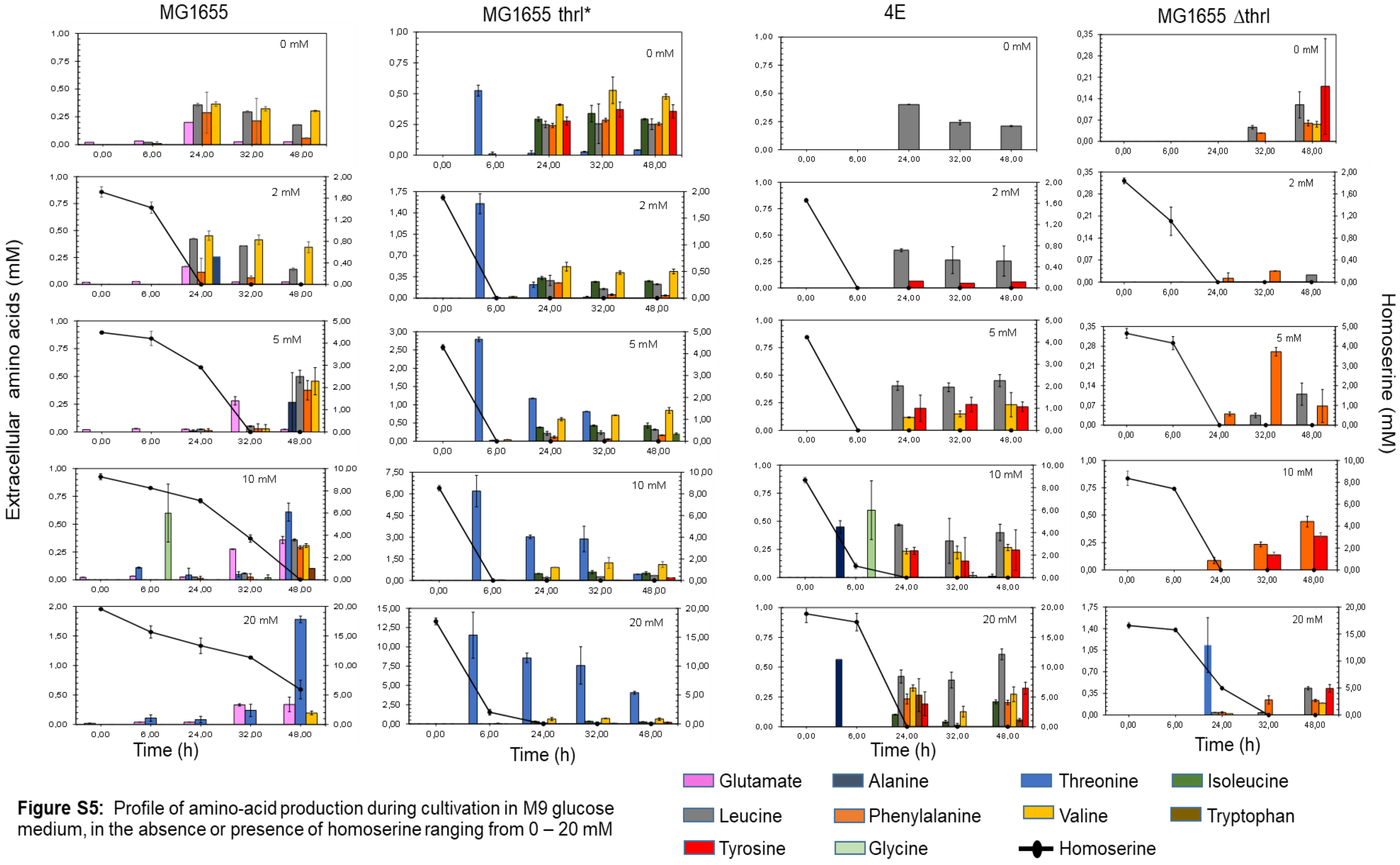

Figure S5: Profile of amino-acid production during cultivation in M9 glucose medium, in the absence or presence of homoserine ranging from 0 – 20 mM

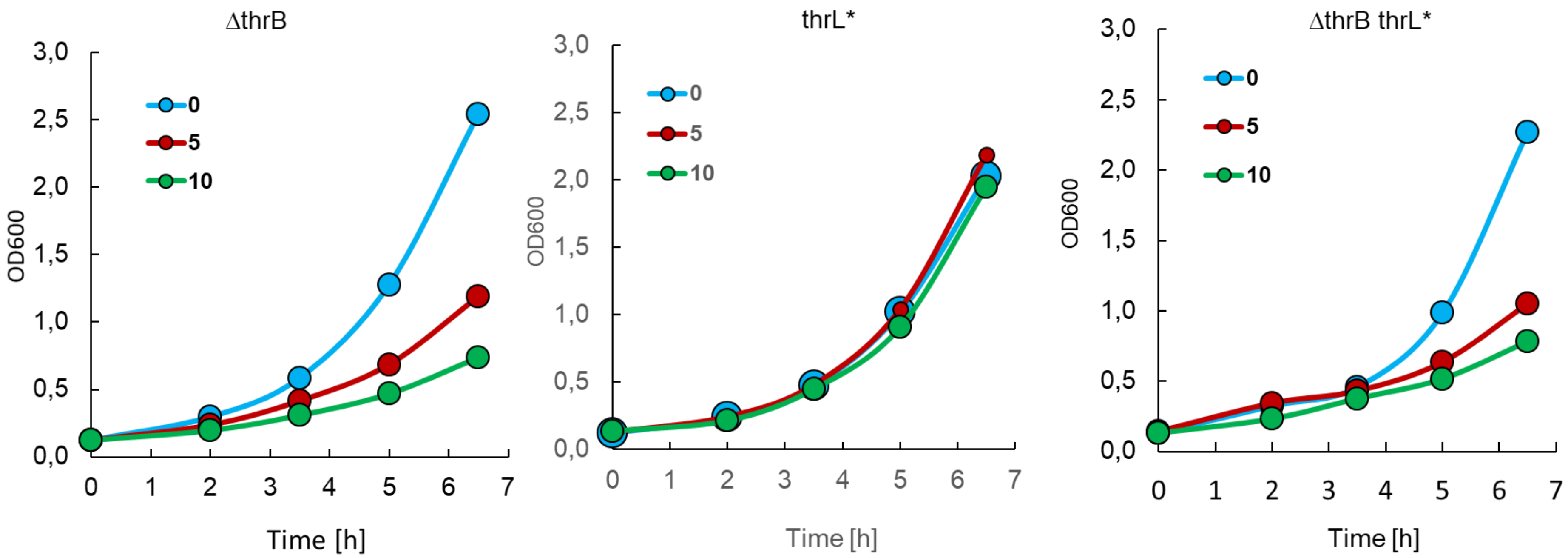

Figure S6 : Sensitivity of thrl* strain to homoserine is restaured upon deletion of *thrB* encoding homserine linase. Growth was carried out in M9 medium at 37°C in the presence of different concentration of L-homoserine as indicated in the figure.
